# Separation, Immobilization, and Biocatalytic Utilization of Proteins by a Supramolecular Membrane

**DOI:** 10.1371/journal.pone.0063188

**Published:** 2013-05-10

**Authors:** Elisha Krieg, Shira Albeck, Haim Weissman, Eyal Shimoni, Boris Rybtchinski

**Affiliations:** 1 Department of Organic Chemistry, Weizmann Institute of Science, Rehovot, Israel; 2 Israel Structural Proteomics Center (ISPC), Weizmann Institute of Science, Rehovot, Israel; 3 Department of Chemical Research Support, Weizmann Institute of Science, Rehovot, Israel; Institute for Frontier Medical Sciences, Kyoto University, Japan

## Abstract

Membrane separation of biomolecules and their application in biocatalysis is becoming increasingly important for biotechnology, demanding the development of new biocompatible materials with novel properties. In the present study, an entirely noncovalent water-based material is used as a membrane for size-selective separation, immobilization, and biocatalytic utilization of proteins. The membrane shows stable performance under physiological conditions, allowing filtration of protein mixtures with a 150 kDa molecular weight cutoff (∼8 nm hydrodynamic diameter cutoff). Due to the biocompatibility of the membrane, filtered proteins stay functionally active and retained proteins can be partially recovered. Upon filtration, large enzymes become immobilized within the membrane. They exhibit stable activity when subjected to a constant flux of substrates for prolonged periods of time, which can be used to carry out heterogeneous biocatalysis. The noncovalent membrane material can be easily disassembled, purified, reassembled, and reused, showing reproducible performance after recycling. The robustness, recyclability, versatility, and biocompatibility of the supramolecular membrane may open new avenues for manipulating biological systems.

## Introduction

Noncovalent materials are adaptive, unlike most conventional covalent systems, rendering them advantageous for a variety of applications. [1]–[12] They are particularly useful for biotechnology, where biocompatible, stimuli-responsive, and self-healing materials have been utilized as artificial tissues, drug delivery devices, and matrices for encapsulation and controlled release of cells. [13]–[19] Yet, a number of important biotechnological applications require high material stability. For instance, membrane filtration of biomolecules employs nanoporous materials that should be stable for long periods of time under the pressure-driven flux of solvent, in the presence of buffer, and at physiological pH and salt concentrations. Membrane filtration is an essential tool in the biotechnological industry and appears to be particularly useful for the purification and concentration of proteins. [20]–[27] Moreover, membranes can be used for immobilization and biocatalytic utilization of enzymes. As enzymes catalyze reactions under very mild conditions, exhibiting efficiency and selectivity largely unmatched by synthetic catalysts, such membrane reactors are emerging components in new, environmentally friendly industrial processes (heterogeneous biocatalysis), which may supplement or replace traditional chemical methods. [28].

Due to high stability demands, virtually all filtration membranes used today are based on polymers or ceramics. [29]–[32] However, while providing remarkable stability, the irreversible bonding in these systems sets limits to the scope of possible adaptive changes, including self-healing, response to external stimuli, and controlled membrane disassembly. In this respect, functionalization of polymer-based membranes with polymeric hydrogel coatings enabled stimuli-responsiveness, for instance temperature- or pH-induced shrinkage/swelling. [33] Importantly, when dealing with biological samples, adaptive changes in the membrane (e.g., for controlled release of biomolecules) should take place under mild conditions to preserve the biomolecules’ structure and function.

Entirely supramolecular membranes can be very advantageous due to the adaptive character of noncovalent bonding. While supramolecular arrays have been used to template the formation of covalent membranes, [34]–[37] only a few supramolecular membranes have been reported that do not require structural reinforcement by covalent crosslinks in the active layer. [38]–[43] For instance, Lu et al. have modified polymer membranes with self-assembling amphiphiles in order to achieve unique rejection properties. [39] Dankers et al. have electro-spun self-assembling molecules to form membranes that functioned as matrices for renal epithelial cells; [43] the performance of this system in pressure-driven filtration was not an object of the study. Others have combined the well-established phase inversion membrane fabrication with self-assembly procedures to create block copolymer micelle-based membranes, [40] some of which exhibit tunable porosity [41] and self-healing properties. [42] However, these membranes employ conventional high molecular weight polymers, and those applied to biological systems require elaborate modifications of the self-assembled material prior to use. [35], [44] To the best of our knowledge, robust biocompatible filtration membranes based entirely on noncovalent bonds between small amphiphilic molecules and operating under physiological conditions have not been reported.

**Figure 1:**
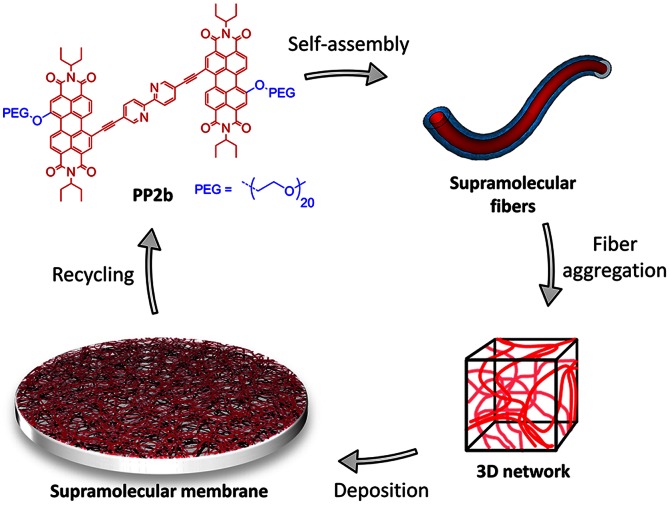
Illustration of membrane fabrication and recycling. Molecular structure of **PP2b**, schematic depiction of supramolecular fibers, 3D network, and membrane. Hydrophobic groups of **PP2b** are located in the fibers’ core (red), whereas their hydrophilic PEG shell (blue) provides a biocompatible interface. Recycling of the membrane is achieved by disaggregation or physical removal of the supramolecular layer from the support, followed by purification, and subsequent reassembly in aqueous solution.

Hydrophobic interactions [45]–[47] between large nonpolar groups of amphiphilic molecules in aqueous solution can be remarkably strong, driving self-assembly towards very stable supramolecular systems. [3], [48]–[50] Based on this idea, we have previously designed bolaamphiphile **PP2b** [51] (Figure 1). Its extended hydrophobic core contains two perylene-3,4,9,10-tetracarboxylic acid diimide (PDI) [52] units. **PP2b** self-assembles in aqueous media into a robust three dimensional (3D) fibrous network, resulting in a stable and multiple-stimuli-responsive material. Recently, we have fabricated **PP2b**-based *supramolecular ultrafiltration membranes* capable of size-selective separation of metal and semiconductor nanoparticles. [38] The membranes are readily prepared within 15 minutes via one-step deposition of an aggregated **PP2b** solution on a microfiltration support. This simple, fast and robust method represents a significant advantage over most fabrication techniques for covalent membranes, which are time-consuming and often require meticulously controlled conditions. Owing to its noncovalent nature, the material is easily disassembled by organic solvent (e.g. ethanol), the retained particles are released, and the membrane material itself can be recycled and reused multiple times.

Are hydrophobically stabilized noncovalent materials such as **PP2b** membranes suitable for biotechnological applications that require robustness? In general, hydrophobic groups can interfere with biomolecules by affecting their stability or by forming hydrophobic surfaces that will stick to proteins. Also, presence of high salt concentrations may significantly alter the structure of supramolecular assemblies, [53], [54] and weak hydrophobic assemblies in particular. Nevertheless, strong noncovalent bonds are sometimes adequate to produce very robust supramolecular arrays. [55]–[58] In this respect, the **PP2b** assembly is based on very strong hydrophobic interactions, preventing exposure of the hydrophobic moieties to bulk water. [1], [59] It is also enclosed by a shell of polyethylene glycol (PEG) groups (Figure 1), [51] which are known to preserve the native structure of proteins and resist undesired biomolecule adsorption (the latter would result in rapid membrane fouling). [60]–[62] Thus, in water, the **PP2b**-based network is potentially both robust and biocompatible, while retaining recyclability due to its noncovalent nature.

The present study demonstrates how the **PP2b-**based membranes can be employed for the manipulation of proteins. First, protein filtration experiments were carried out to characterize the membranes’ performance in bioseparation of protein mixtures, and to determine its molecular weight cutoff. Next, its biocompatible properties were utilized towards immobilization of enzymes that were used in heterogeneous biocatalysis. Such membrane/immobilized enzyme arrays bear great potential to carry out biocatalytic reactions under continuous flow of reactants (i.e. the enzyme’s substrate) that are converted “en passant” into a desired product, combining biocatalytic reaction and catalyst removal in a single step. [63]–[66] As described herein, the **PP2b** membranes are versatile for the manipulation of proteins, allowing recycling of the membrane material and retrieval of proteins. To the best of our knowledge, this is the first report of a noncovalent membrane that performs separation, immobilization and biocatalytic utilization of proteins, thus demonstrating the great potential of hydrophobically stabilized noncovalent materials in biotechnology.

## Results and Discussion

### 1. Fabrication and Characterization of PP2b Supramolecular Membranes

**Figure 2:**
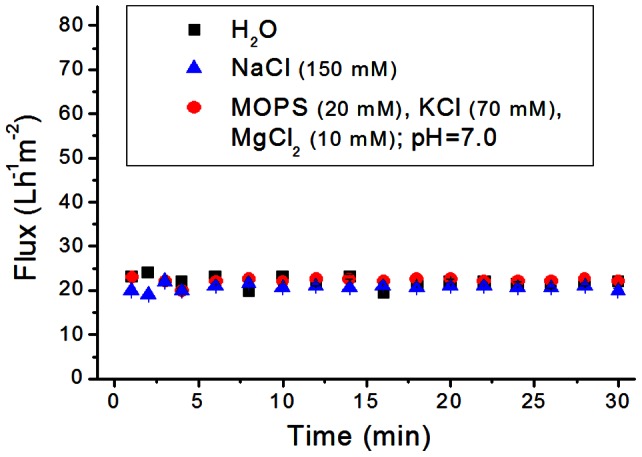
Stable flux of aqueous solutions over time. Flux of water, NaCl(aq), and MOPS buffer solution through a freshly prepared supramolecular membrane.

**Figure 3:**
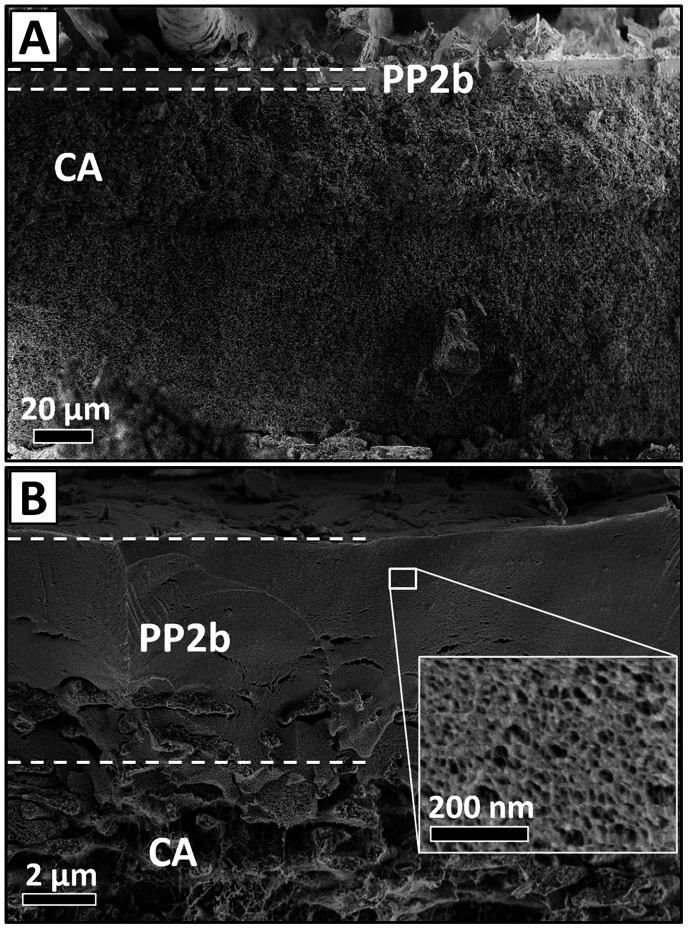
Membrane cross-section. Cryo-SEM images of the supramolecular PP2b membrane on the cellulose acetate (CA) support, which had been permeated by MOPS buffer solution.

The membranes used in this study were fabricated by depositing 1 ml of an aggregated solution of **PP2b** (0.5 mM) in water on a cellulose acetate (CA) support (0.45 µm pore size; effective filtration area: 5.7 cm^2^), analogously to the reported procedure (see Materials and Methods). [38] Hence, each supramolecular membrane was composed of 1.5 mg **PP2b**. Importantly, the membranes remain robust under physiological conditions, e.g. aqueous NaCl (150 mM), and 3–(N-morpholino) propanesulfonic acid (MOPS) buffer solution, as revealed by monitoring the solution flux through the membrane over time (Figure 2). The flux was virtually identical for different aqueous solutions and remained constant during the experiment (30 min), indicating that neither membrane disassembly nor significant compression of the material took place. Fulfilling this first requirement ensured that the membranes are sufficiently stable for typical laboratory-scale filtrations of biological systems. During the course of our experiments, supramolecular membranes showed reproducible performance for filtration and sustained continuous solvent flux for up to 6 hours (see below). The flux of 20 Lh^−1^m^−2^ at 0.8 bar transmembrane pressure was somewhat lower than usual commercial ultrafiltration membranes with similar rejection properties, which exhibit typical fluxes of 50–100 Lh^−1^m^−2^. [67] The membrane thickness was approximately 6 µm, as revealed by cryogenic scanning electron microscopy (cryo-SEM) (Figure 3). Higher magnification of its cross-section indicates that the material retains its nanoporous structure under physiological conditions (Figure 3B, inset), which is critical for size-selective bioseparations.

### 2. Separation Performance

A mixture of six purified proteins was used to carry out filtration experiments: 1. N-terminal domain of EIIBCA-Bgl residues 2–84 (EIIBCA), 2. *In silico* designed Kemp eliminase (KE70), [68], [69] 3. L-carnitine dehydratase (LCD), 4. L-Fuculose-1-Phosphate Aldolase (Aldolase), 5. Citrate Synthase (CS), 6. Bovine Serum Albumin (BSA). This protein mixture represents a broad range of masses, making it suitable for characterization of the filtration cutoff. The proteins were dissolved in MOPS buffer solution (MOPS, 20 mM; KCl, 70 mM; MgCl_2_, 10 mM; pH = 7.0) with an overall protein concentration of 1.8 mg/ml (0.3 mg/ml for each protein). In order to evaluate the actual size of the individual proteins in solution, they were each analyzed by gel filtration chromatography (GFC) (Figure S3 in Text S1), and their hydrodynamic diameters (D_h_) were determined using dynamic light scattering (DLS) (Figure S4 in Text S1). We note that not all proteins in the mixture were monomeric; CS exhibited a molecular weight of approximately 288 kDa (from GFC), corresponding to its well-known hexameric form (301 kDa), [70], [71] Aldolase was a tetramer, [72] and the commercially obtained BSA was oligomeric (≥400 kDa, determined by GFC). These findings are consistent with DLS measurements, showing a size increase in the order EIIBCA<KE70< LCD<Aldolase<CS<BSA (Table 1).

**Table 1:** Size-related values of the proteins: calculated molecular weight (MW), hydrodynamic diameter (D_h_), and molecular dimensions estimated from X-ray structures.

Protein	MW [kDa]	D_h_ [nm]	Protein X-ray structure dimensions [nm] (PDB code)
**EIIBCA**	8.7	3.4	n.a.
**KE70**	29	5.4	5.0×4.1×3.8 *(3Q2D)*
**LCD**	92	7.2	n.a.
**Aldolase**	158	8.2	7.0×7.0×5.5 *(1DZU)*
**CS (hex.)**	301	11.4	13.1×12.3×8.2 *(homologous to 1NXG)*
**BSA (olig.)** a	≥400	12.8, 57.8	n.a.

a BSA had a bimodal size distribution.

**Figure 4:**
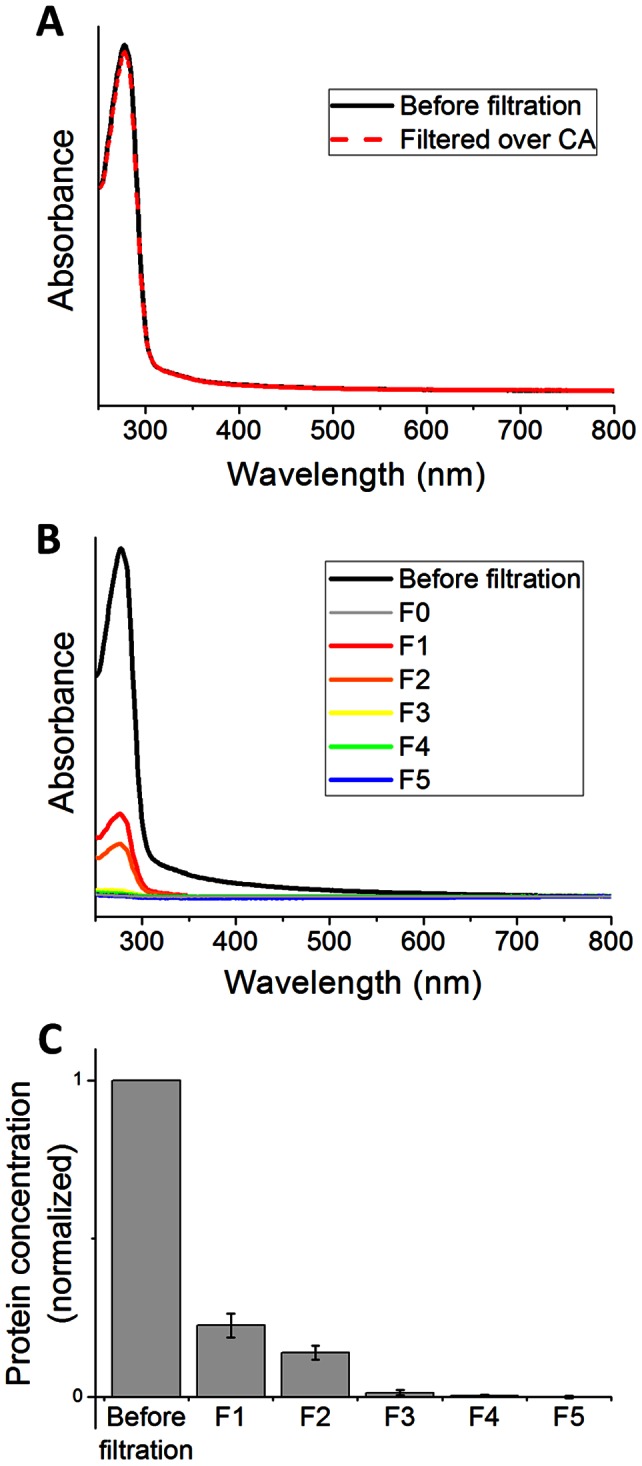
Filtration of protein mixtures. (A) UV/Vis spectra of the protein mixture before and after filtration over the pristine CA membrane. (B) Representative UV/Vis spectra of the protein mixture before filtration through the supramolecular membrane, the filtrate (collected in 5×1.5 ml fractions, F1–F5), and filtered buffer solution as a reference (F0) (C) Total protein concentration in the filtrate fractions F1–F5 as compared to the feed solution, determined from absorbance at 280 nm. Error bars correspond to the standard deviation of 5 independent filtration experiments.

Filtration experiments were performed employing a flow of the protein mixture (1.5 ml) in aqueous MOPS buffer solution through a freshly prepared supramolecular membrane at 0.8 bar transmembrane pressure. As the feed solution was passing the membrane, the filtrate was collected (1.5 ml, F1). Subsequently, additional clean buffer solution (6 ml) was filtered to rinse out remaining proteins from the membrane, and another four fractions were collected successively (4×1.5 ml, F2–F5). The dead volume of the filter chamber was 0.8 ml. UV/Vis spectra of all fractions were recorded (Figure 4B, Figure S7 in Text S1). Relative protein concentrations for the collected fractions were quantified via absorbance at 280 nm. The fractions did not exhibit any absorption feature in the visible spectrum, showing that no detectable amount of **PP2b** (λ_max,1_ = 393 nm, λ_max,2_ = 538 nm) was leaching out of the membrane during filtration. The first two filtrate fractions (F1 and F2) contained considerable amounts of protein; F3 contained only minor amounts, whereas F4 and F5 were virtually protein-free (Figure 4C). Altogether, 38±4% of the proteins passed the membrane. In a control experiment, the protein mixture was also passed through the pristine CA membrane without the **PP2b** layer. As confirmed by UV/Vis spectra of the filtrate, the CA membrane itself did not retain any proteins (Figure 4A), showing that the retention is due to the supramolecular membrane only and not caused by adsorption on the support.

Importantly, proteins that were retained on the supramolecular membrane could be partially recycled by dispersing the used membrane in buffer solution (using a vortex mixer), followed by removing the **PP2b** supramolecular material via centrifugation. The resulting supernatant contained retained proteins while the pellet contained **PP2b** together with some proteins that could not be retrieved. The mechanical dispersion of the supramolecular membrane is enabled by its reversible noncovalent bonding, representing a mild alternative to common release mechanisms in covalent polymers, such as pH and temperature-induced shrinkage/swelling. [32], [33] Notably, the enzymatic activity of proteins recycled from the **PP2b** membrane is fully retained (see below).

**Figure 5:**
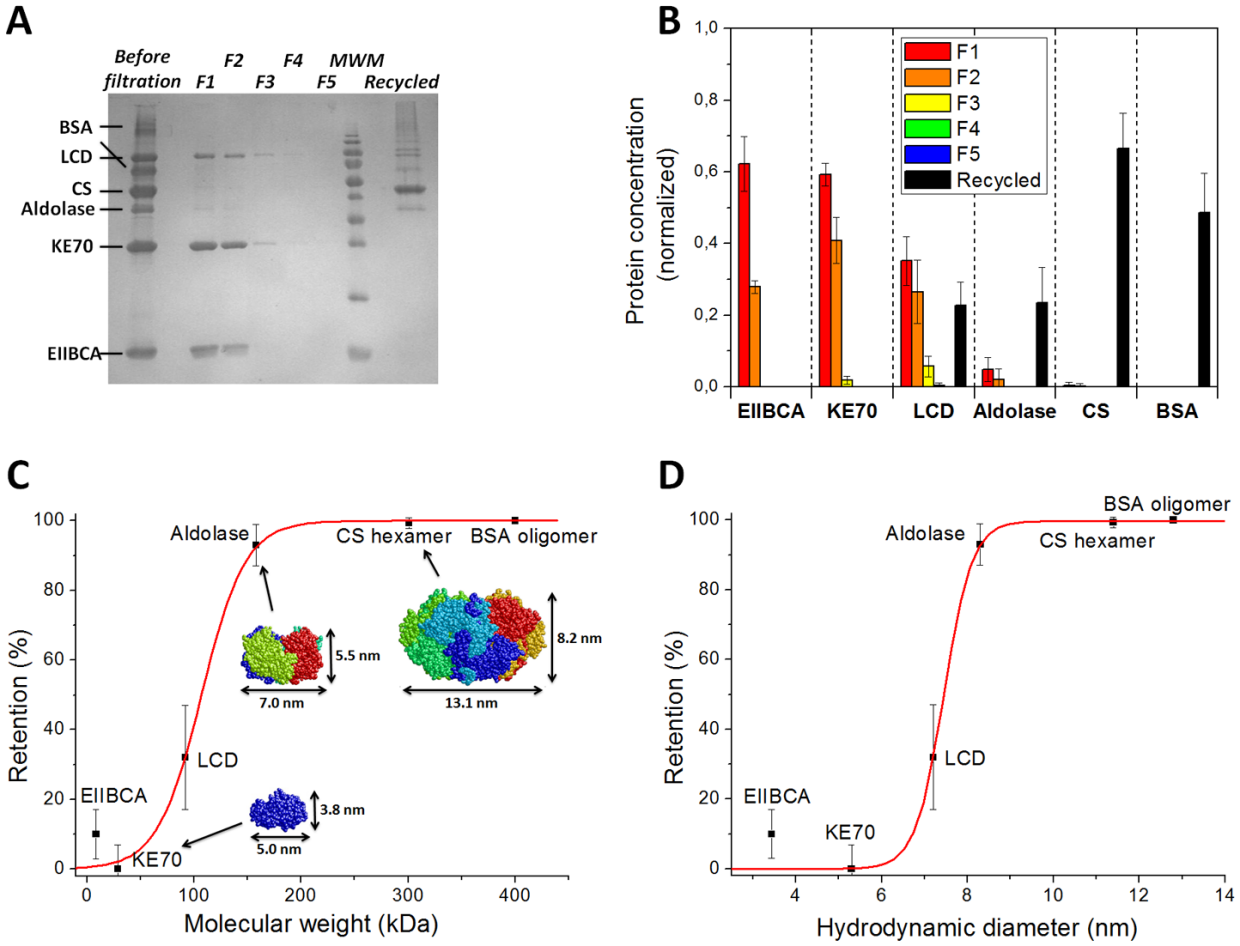
Separation of protein mixtures. (A) Typical SDS-PAGE used for densitometric quantification of individual protein concentrations. MWM = molecular weight marker (170, 130, 95, 72, 55, 43, 34, 26, 17, 11 kDa). (B) Concentrations of individual proteins in fractions F1–F5 (normalized with respect to the non-filtered solution), and recycled proteins. (C) Protein retention against molecular weight (black data points) and sigmoid fit (red curve). Protein structures of KE70 (PDB;3Q2D), Aldolase (PDB;1DZU) and CS hexamer (PDB;1NXG) are shown. (D) Dependence of protein retention on the hydrodynamic diameter (black data points) and sigmoid fit (red curve). All error bars represent the standard deviation of 5 independent filtration experiments.

The filtration experiment results were analyzed by sodium dodecyl sulfate polyacrylamide gel electrophoresis (SDS-PAGE) (Figure 5A). Individual protein concentrations in filtrate and retentate were estimated densitometrically from the gel (Figures S5, S6 in Text S1). Filtration experiments were repeated five times using independently deposited membranes, which showed consistent separation performance (Figure 5, Figures S6, S7 in Text S1). Figure 5C shows the retention (the percentage of proteins filtered from the solution) as a function of the molecular weight. It is known for the **PP2b** membrane that retained particles are captured in the interior of the membrane rather than on the surface (depth filtration). [38] In depth filtration, the average observed pore sizes of the membrane are usually an order of magnitude larger than the cutoff value, since particles are captured at constrictions within the membrane or by adsorption on pore surfaces. [73] The retention of proteins in the **PP2b** membrane is clearly size-dependent and follows a typical sigmoid curve, indicating that size-selective capture (e.g. through mechanical sieving) plays a major role in the filtration process, rather than specific (e.g. electrostatic) protein adsorption. However, adsorption might play a minor role, for instance in the retention of small amounts (∼10%) of EIIBCA. The molecular weight cutoff, defined as the theoretical weight of a molecule with 90% retention, was determined from the retention curve (Figure 5C), as **150 kDa**. In terms of hydrodynamic diameter, the membrane cutoff is **8 nm** (Figure 5D), which is somewhat larger than the previously reported 5 nm cutoff for spherical gold nanoparticles. [38] Yet, the latter value refers to the nanoparticles’ rigid core, which does not include organic capping agent, counter ions, and hydration shell. The available protein crystal structures [69], [70], [72] reveal that the actual protein dimensions are significantly smaller than the corresponding hydrodynamic diameters (Figure 5C, Table 1). For instance the smallest dimension of KE70 is only 3.8 nm, enabling it to pass the membrane, whereas the smallest dimension of Aldolase is 5.5 nm, resulting in >90% retention. Thus, good agreement with previous nanoparticle filtration experiments is observed. [38] We note that size-selective retention in a membrane can depend on shape and deformability of filtered particles. [73].

Figure 5B details the concentration of the proteins in each of the filtrate fractions and that of the retained proteins recycled from the membrane. Notably, permeation of the three smaller proteins EIIBCA, KE70, and LCD, showed a size-dependent trend: The smallest (EIIBCA) passed the membrane with a short time delay and was completely collected in F1 and F2, whereas the larger (KE70) exhibited a longer delay, and small amounts were detected in F3 as well. Finally, the largest of these three (LCD) was found in considerable amounts in F3, and its traces were detected in F4. For the retained proteins, the efficiency of recycling was not equal. For example, about 66% of CS was regained from the filter, whereas only 25% of retained Aldolase could be recycled, possibly due to aggregation or irreversible entrapment within the supramolecular fibers of the membrane material. The membrane itself can be disassembled in water/ethanol (2∶3, v/v) and cleaned from contaminations via chromatography over a short silica column (see Figure S2 in Text S1, showing the ^1^H-NMR of **PP2b** before and after recycling). It is easily re-assembled and re-used as a membrane, having similar thickness and *identical separation performance* as compared to the membranes prepared from non-recycled **PP2b** (Figure S8 in Text S1).

Facile recycling and reproducible separation performance after recycling can be regarded as a direct result of the noncovalent nature of the membrane material: its nanoscopic structure (i.e. the three dimensional network of supramolecular fibers) [51] is encoded at the molecular level, allowing simple self-assembly and deposition procedures.

**Figure 6:**
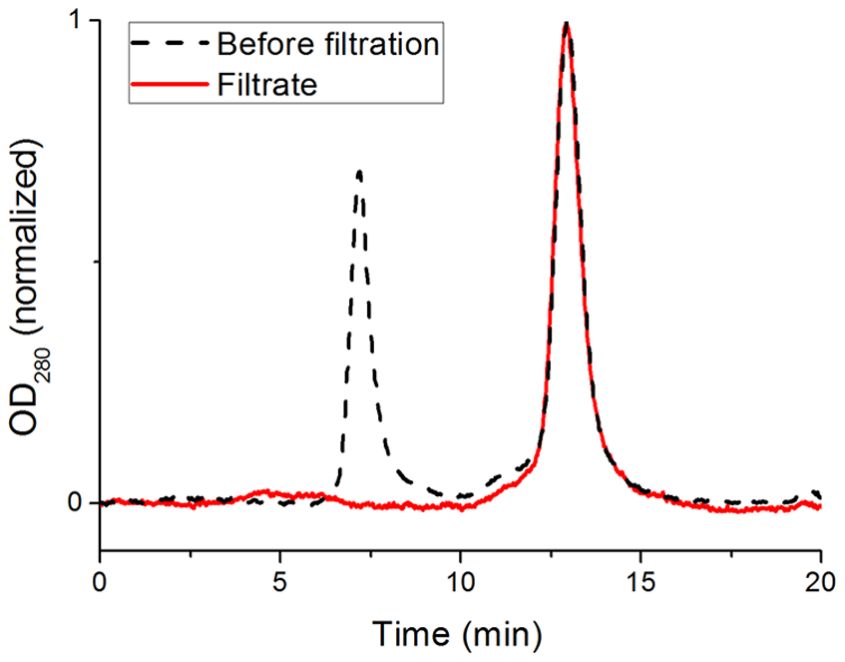
Purification of BSA. Gel filtration chromatogram of a mixture of BSA oligomers and monomers before filtration (black trace), and its filtrate (red trace). Filtration quantitatively removes BSA oligomers (≥ 400 kDa, retention time: 7 min) from the mixture. Smaller BSA aggregates (retention time: 11–12 min) are removed as well. The filtrate contains pure monomeric proteins (∼67 kDa, retention time: 13 min).

The cutoff value of the supramolecular membrane is in the upper range of commonly used ultrafiltration membranes in biotechnology, allowing the retention of large proteins, nucleic acids, lipids and other large lysate components. [20], [21], [23] We emphasize that the described filtration characteristics can be particularly useful for the rapid separation of protein monomers from protein aggregates. In order to specifically demonstrate monomer/aggregate separation, oligomeric BSA was mixed with specially prepared monomeric BSA and the mixture was filtered over the **PP2b** supramolecular membrane. Due to their small size, the protein oligomers (D_h_ ∼ 12.8 nm) cannot be removed by a standard desktop centrifuge (20800 g). However, as revealed by GFC, the supramolecular membrane efficiently removed oligomeric BSA from the mixture, resulting in a filtrate of pure BSA monomer (Figure 6).

### 3. Biocatalytic Utilization of the Supramolecular Membrane

**Figure 7:**
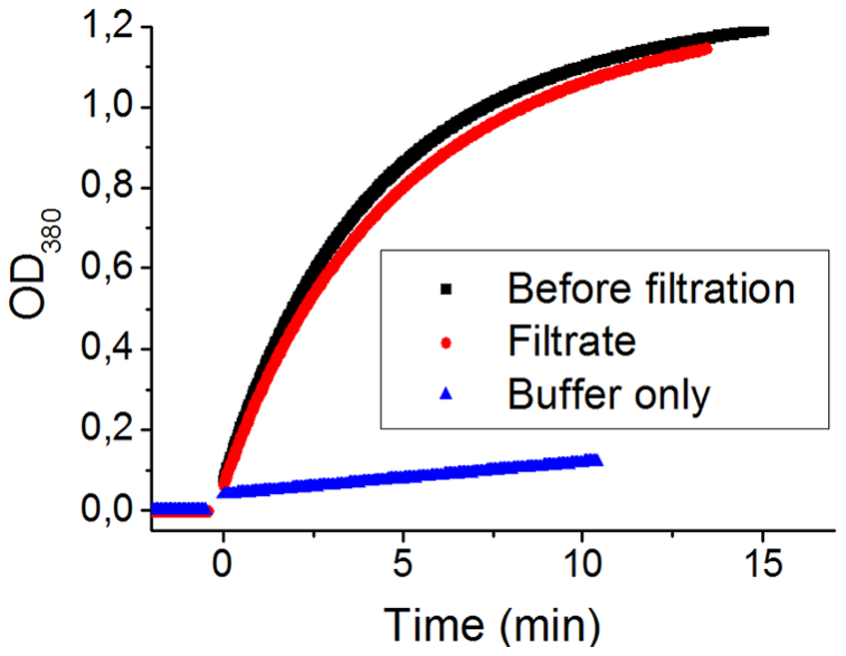
Kinetics of the KE70 activity. Change in absorbance at 380 nm before filtration, after filtration, and of neat buffer solution, following addition of 5-Nitrobezisoxazole (at t = 0 min).

In order to examine whether the filtration process affected protein function, we tested the enzymatic activity of KE70 at 25°C before and after filtration. Using a standard assay, [68] the activity of the enzyme solution before filtration, in the filtrate, and in neat buffer solution (background reaction) was determined by measuring the kinetics of the enzyme-catalyzed isomerisation of 5-Nitrobezisoxazole via absorbance of the product at 380 nm (OD_380_) (Figure 7, Figure S10 in Text S1). Due to dilution with residual buffer solution in the filter chamber, the concentration of KE70 in the filtrate was 90% with respect to the feed solution. In comparison, its relative activity was 88%, revealing a near-quantitative (∼98%) conservation of activity (see section S10, Figure S9, and Table S1 in Text S1).

In addition to KE70, we also investigated the enzymatic activity of hexameric CS after filtration. In contrast to KE70, CS is completely retained and can be recycled from the membrane. In case of retained enzymes, their structure and function might be affected by the extensive contact with the supramolecular material, and by the subsequent recycling procedure. CS activity before filtration and after recycling from the membrane was quantified at 25°C according to an activity assay based on a literature procedure: [74] measuring the kinetics of Citrate formation from Oxaloacetate and Acetyl Coenzyme A (Acetyl-CoA) by detecting the indicator of the reaction, 2-nitro-5-thiobenzoate (TNB) at 412 nm (OD_412_) (Figure S11 in Text S1). Taking into account changes in concentration (determined via Bradford test), [75] the kinetic measurements revealed quantitative retention of enzymatic activity of CS, underscoring the membrane’s biocompatibility and the mild conditions of enzyme release from the membrane (see section S11, Tables S2, S3 in Text S1).

Our experiments show that filtration of large enzymes results in their immobilization within the supramolecular network, keeping them exposed to the flux of solvent and solutes. Such an array (membrane/immobilized enzyme) may represent a versatile system to carry out biocatalytic reactions in a heterogeneous manner. Addressing this concept, we investigated the enzymatic activity of membrane-immobilized β-Galactosidase (β-Gal, 465 kDa). β-Gal is significantly larger than the membrane cutoff, resulting in near-quantitative retention. It is ubiquitous in nature and widely utilized in molecular biology, catalyzing the hydrolysis of the glycosidic bond of β-Galactopyranosides. We note that β-Gal and many other hydrolases do not require presence of coenzymes, making them the most relevant enzyme class for biotechnological applications in industry, [28] e.g., β-Gal has wide application in the dairy industry for the production of low-lactose milk. [28].

**Figure 8:**
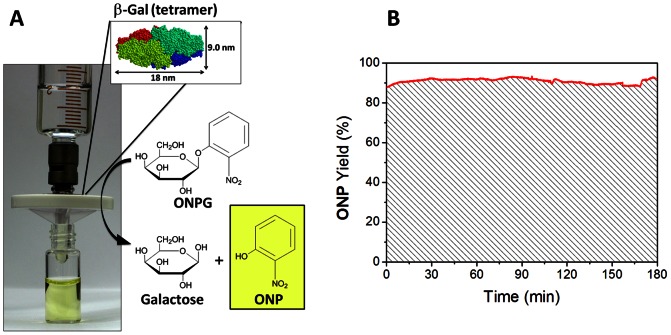
Heterogeneous biocatalysis in the supramolecular membrane using immobilized β-Gal. (A) Hydrolysis of ONPG into Galactose and ONP. (B) Yield of ONP as a function of time during several hours of continuous flux of substrate.

For immobilization, 1.5 ml of β-Gal (0.2 mg/ml) was filtered over a freshly prepared supramolecular membrane, and rinsed with 7.5 ml of clean buffer solution. Once the enzyme was retained on the membrane, the solution flux slightly dropped from 20 L h^−1^ m^−2^ to 17 L h^−1^ m^−2^. Subsequently, a typical activity assay solution [76] containing the substrate o-Nitrophenyl β-D-galactopyranoside (ONPG, 0.05 mg/ml) was passed through the membrane. As the colorless ONPG feed solution passed through the membrane it turned yellow, indicating the β-Gal-catalyzed conversion of ONPG into o-Nitrophenol (ONP, λ_max_ = 420 nm) (Figure 8A). In order to quantify the conversion and to study performance under constant substrate flux for prolonged periods of time, the filter was connected to a UV/Vis flow cuvette, and the absorbance of the filtrate at 420 nm was recorded (Figures S14, S15 in Text S1). Importantly, the reaction showed stable conversion over several hours of uninterrupted substrate flow with an average yield of ∼90% ONP (Figure 8B).

Small amounts of enzyme were observed to leach out of the membrane over time, as expected in immobilization that does not involve covalent attachment of the enzyme to the support. [66] However, enzyme leaching was very low (decreasing from 0.24% to 0.046% activity with respect to the original enzyme solution; see section S15, Figure S16, Table S5 in Text S1) and did not influence the overall reaction yield. Emphasizing its remarkable robustness and biocompatibility, the supramolecular membrane/embedded enzyme array sustained stable operation under a constant flux of solution for overall 6 hours (incl. preparation, rinsing steps, and test of enzyme leaching), out of which 3 hours were dedicated to biocatalytic substrate conversion.

**Figure 9:**
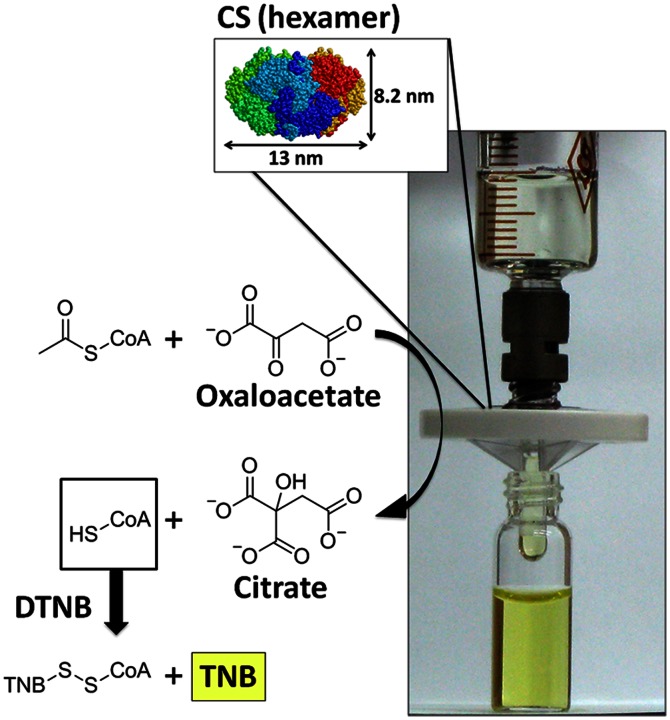
Conversion of Oxaloacetate and Acetyl-CoA into Citrate and HS-CoA over CS immobilized in a PP2b supramolecular membrane. HS-CoA reacts with DTNB in the assay solution to release the indicator of the reaction, TNB (λ_max_ = 412 nm). The color change from clear reactant feed solution to yellow filtrate indicates biocatalytic activity of the immobilized enzymes.

In an additional experiment, hexameric CS was immobilized and tested for its biocatalytic activity. While β-Gal facilitates the degradation of a complex molecule (ONPG) into smaller parts (Galactose, ONP), CS represents a distinctly different case: a simple precursor (Oxaloacetate) is converted into a more complex product (Citrate), requiring the presence of a coenzyme (Acetyl-CoA). CS immobilization was performed by simply filtering 2 ml enzyme solution (0.3 mg/ml) over a freshly prepared supramolecular membrane, followed by rinsing with 10.5 ml clean buffer solution. We employed a common assay solution [74] to test activity of immobilized CS. When running the mixed solution of Oxaloacetate, Acetyl-CoA, and 5,5′-Dithiobis(2-nitrobenzoate) (DTNB) through the membrane, biocatalytic formation of Citrate was indicated by the yellow color of the filtrate, which resulted from the subsequent reaction between HS-CoA and DTNB, forming TNB (λ_max_ = 412 nm) (Figure 9). Thus, conversion of Oxaloacetate into Citrate under constant flow of substrate and coenzyme was readily achieved. Similar to β-Gal, leaching of CS was low (∼0.23% activity with respect to the original enzyme solution; see section S13, Figures S12, S13, Table S4 in Text S1).

Overall, immobilization on the supramolecular membranes is versatile and applicable to various enzymes provided that they are large enough to be retained. In order to extend the method to smaller enzymes, one can use standard procedures to enlarge the enzyme without affecting its activity, either by fusing the enzyme to a large inactive protein, or by formation of cross-linked enzyme aggregates (CLEAs) prior to filtration. [64], [66], [77] Since the design of effective immobilization techniques has been described as one of the main obstacles for industrial-scale biocatalysis, [78] we note that enzyme entrapment in the supramolecular membrane is accomplished in a simple filtration step within 20 minutes. The reactant conversion can be controlled by adjusting the amount of enzyme deposited on the supramolecular membrane. Having a substantial thickness of ∼6 µm the noncovalent matrix functions as a depth filter, allowing very high enzyme loading (e.g. 0.4 g enzyme/1 g **PP2b** membrane) without membrane clogging. In contrast, most commercial ultrafiltration membranes retain particles on their surface (screen filtration), which enables high solution flux but limits the membranes’ capacity for loaded enzymes. [73] Since biocatalysis in membrane reactors takes place within the short time of the substrate’s passing through the membrane layer, high enzyme loading is important in order to achieve satisfying reaction yields. Heterogeneous biocatalysis also offers an elegant method to facilitate more complex cascade reactions, if two or more enzymes are immobilized in a sequential manner. [66], [79] As membrane fabrication and enzyme immobilization are carried out by simple deposition steps, preparation of complex layered structures with alternating membrane/enzyme arrays [79] can be feasible.

Employing a *fully noncovalent* membrane reactor for enzyme biocatalysis has several advantages: no synthetic modification or covalent attachment of the enzyme to a stationary phase is necessary. Moreover, enzymes can be easily retrieved from the membrane by disassembly of the noncovalent membrane material, which is important considering the high cost of enzyme synthesis. These factors represent a significant advantage over the commonly employed covalent attachment of enzymes to a support, which is a slow and complex (multistep) process, can affect enzyme activity, and prohibits simple enzyme retrieval. [28].

### Conclusions

In summary, we have demonstrated the application of a noncovalent membrane for separation, immobilization and biocatalytic utilization of proteins. To the best of our knowledge, this is the first example in which these functions were achieved using an entirely noncovalent water-based material. The membrane exhibits stable performance under physiological conditions, allowing size-selective separation with a 150 kDa molecular weight cutoff (∼8 nm hydrodynamic diameter cutoff). Importantly, separated proteins - both passing and retained - remain functionally active, underlining the biocompatibility of the noncovalent material and separation process. Although the membrane operates under rather low pressure (0.8 bar) and flow rate (20 Lh^−1^m^−2^), it has several significant advantages over conventional polymer membranes: 1) Membrane fabrication via deposition of self-assembled fibers is significantly easier and faster than common polymer processing methods. [29] 2) The noncovalent material can be disassembled under benign conditions, e.g. in case of physical damage, clogging, or for intentional protein release. Subsequently, the membrane material can be easily purified, reassembled, and reused multiple times, [38] thus being fully recyclable and cost-efficient. 3) Retained proteins can be retrieved using simple procedures.

Facile membrane fabrication followed by deposition of large proteins represents a simple and fast alternative to covalent enzyme immobilization. Such membrane-immobilized enzymes can be utilized in heterogeneous biocatalysis, which may allow “green” industrial processes and development of enzyme-based sensors. The noncovalent nature of the supramolecular membranes is expected to enable further modifications [14] and realization of more complex tandem reactions. Overall, we demonstrate that self-assembled noncovalent materials based on strong hydrophobic interactions are advantageous for biotechnological applications, where a combination of facile fabrication, robustness, and adaptivity provide new opportunities for manipulating biomolecules.

## Materials and Methods

### Materials and Methods

Solvents and reagents were purchased from commercial sources and used as received, unless otherwise specified. Synthesis of **PP2b** was reported previously. [51] For all aqueous mixtures double-distilled water was used (Barnstead NANOpure Diamond™ water system). Cellulose acetate (CA) syringe filters were purchased from Whatman (Puradisc FP 30/0.45 CA-S). Protein crystal structures were obtained from the PDB (http://www.rcsb.org) and visualized with *RasMol* (v. 2.7.4.2.). [80], [81].

^1^H-NMR spectra were recorded at room temperature on a 300 MHz spectrometer (Bruker).

UV/Vis absorption measurements were carried out on a Cary-5000 spectrometer (Varian).

MALDI-TOF mass spectrometry was carried out using a REFLEX™ reflector time-of-flight instrument with SCOUT™ multiprobe (384) inlet. Chloroform was the solvent for all samples analyzed by mass spectrometry.

GFC was performed on an AKTA purifier using an analytical Superdex 200 10/300 GL column (GH Healthcare). The column was equilibrated with buffer containing 20 mM Tris pH 7.5 and 100 mM NaCl. The proteins were injected individually and their migration was compared to commercial molecular weight markers.

DLS was carried out on a Viscotek 802 DLS (Malvern Instruments). Samples were measured in a 12 µl quartz cuvette. Data analysis was performed using OmniSIZE (v. 3,0,0,292). 544 correlator channels were used. The data acquisition time was set to 5–10 seconds. A mass-weighted size distribution was determined from a single correlation function, which was obtained from the average of at least 8 experiments.

SDS-PAGE was performed using 15% gels and stained with Coomassie blue. 5 µl sample buffer was added to 15 µl of each protein sample (0.3 mg/ml) from which 15 µl was applied to each lane. The individual bands were quantified using the QuantityOne volume analysis package (BioRad version 4.4.0). The bands were analyzed densitometrically via the absorbance of UV light integrated over selected band areas. The background absorbance subtraction was set to “local”, which reduced the error arising from uneven background brightness.

Cryo-SEM sample preparation involved the high pressure freezing (HPF) technique, which is designed to minimize ice crystal formation. [82]–[85] For this purpose, a ∼1×1 mm small rectangle was cut out from the membrane and placed inside the inner cavity of an aluminium planchette (size = 3.0 mm (diameter)×0.5 mm (thickness); inner cavity = 2.0 mm (diameter)×0.15 mm (depth)). The vacant space in the cavity was filled with 1-hexadecene and it was capped with the flat side of another aluminium planchette. HPF was carried out using a Bal-Tec HPM 010 high pressure freezing machine. Subsequently, the sandwich was transferred into a Bal-Tech BAF 060 freeze fracture system where it was fractured with a pre-cooled razorblade and solvent was allowed to sublime (−105°C, 60 min). Subsequently, it was coated with Pt/C, employing double axis rotary shadowing (DARS). Images were taken in a Zeiss Ultra 55 cryogenic scanning electron microscope operated at 1–5 kV with an aperture size set to 10 µm, utilizing an in-lens secondary electron detector.

### Protein Mixture for Filtration Experiments

The protein mixture was prepared by dissolving 0.51 mg of each of the following proteins (from stock solutions) in MOPS buffer solution (1.7 ml): EIIBCA, KE70, LCD, Aldolase, CS, BSA (protein concentrations were determined relative to BSA using Bradford reagent [75]). The solution was vortexed and centrifuged for 5 min at 20800 g, in order to assure complete protein dispersion. 1.5 ml of the clear protein mixture was used for filtration experiments within one hour.

### Preparation of PP2b with Optimized PEG Length

**PP2b** was synthesized following our previously reported procedure. [51] Silica column chromatography (eluent: CHCl_3_/MeOH, 0–6% MeOH gradient) was used to fractionate the material into two batches with different average PEG lengths (**PP2b(s)** and **PP2b(l)**). As determined by MALDI-TOF mass spectrometry, **PP2b(s)** (m/z[M+Na^+^] = 2973.46, found: 2973.51) is furnished with PEG chains of 18.5 units length on average (Figure S1A in Text S1) and **PP2b(l)** (m/z[M+Na^+^] = 3150.57, found: 3150.85) carries PEG chains with 20.75 units on average (Figure S1B in Text S1). While aggregates of pure **PP2b(s)** in water are not stable over time and begin to precipitate from the solution shortly after preparation, **PP2b(l)** forms a stable homogeneous aqueous solution, but its supramolecular structures are not suitable as ultrafiltration membrane, since they are not efficiently retained by the CA support membrane. The optimum average PEG-length in **PP2b** was adjusted by mixing **PP2b(s)** and **PP2b(l)**. The mixture of 0.5 mg (0.17 µmol) **PP2b(s)** and 1.0 mg (0.32 µmol) **PP2b(l)** was found to produce a stable and sufficiently robust supramolecular solution, which was used for fabrication of supramolecular ultrafiltration membranes.

### Self-assembly of PP2b

An aqueous **PP2b** supramolecular solution (0.5 mM) was prepared similarly to the previously reported procedure: [38] In a typical experiment, 26 mg (8.8 µmol) of **PP2b(s)** and 53 mg **PP2b(l)** (16.8 µmol) were dissolved in THF (2.56 ml). Then water (48.6 ml) was added, followed by vigorous shaking of the solution and sonication for 5 min. After aging for 2 h, THF was removed by evaporating ∼15–20% of the solution at r.t. in high vacuum. The solution was refilled with water to its original volume (51 ml); it was shaken, sonicated for 5 min, and aged for at least 12 h. The mixture was used as a stock solution for preparation of many supramolecular membranes. It was stored at 4°C and could be used for ∼2 weeks, producing membranes with consistent separation performance. During storage for prolonged periods of time (>2 weeks), significant amounts of **PP2b** began to precipitate out of the solution, making it necessary to dry and re-assemble the material as described above.

### Fabrication of Supramolecular Membranes

Membrane fabrication was similar to previously reported. [38] **PP2b** supramolecular solution (1 ml) was diluted in water (2 ml) and the mixture was filtered over a syringe filter (Whatman Puradisc FP 30, CA; effective filtration area: 5.7 cm^2^; pore size: 0.45 µm). During filtration, **PP2b** was deposited on the CA support, as seen by the color change from dark purple feed solution to colorless filtrate, and formation of a dark purple layer on the CA support. The transmembrane pressure was created by opening the gauge to a pressurized nitrogen gas cylinder. It was slowly increased to 0.8 bar during deposition of **PP2b**. Care was taken to avoid air bubbles in the filter chamber, which would hamper the formation of a continuous supramolecular layer. Subsequently, water (2 ml) was filtered through the membrane, which ensures uniform packing of the supramolecular material. [38] In order to adjust pH and ionic strength in the filter chamber to the pH and ionic strength of the protein solution that was to be filtered, neat buffer solution (3 ml) was run through the membrane. The filtrate of that buffer (F0, 1.5 ml) was collected as a reference for UV/Vis spectroscopy. The filter housing was kept filled with buffer solution and the supramolecular membrane was used directly for filtration experiments.

### Filtration Experiments

The protein mixture (1.5 ml) was filtered over a freshly prepared supramolecular membrane. Then, 6 ml of protein-free MOPS buffer solution was filtered in order to rinse out residual proteins from membrane and filter housing. Fractions were collected (5×1.5 ml, F1–F5). To test reproducibility of the filtration outcome, the experiments were carried out five times, each time using another freshly prepared membrane. For consistency, the same transmembrane pressure of 0.8 bar was used in all experiments. The collected fractions were analyzed by UV/Vis (Figure S7 in Text S1) and SDS-PAGE (Figure S6 in Text S1). The reported values for protein concentrations and protein retentions (Figure 4, Figure 5) are mean values with error bars representing their standard deviations.

### Recycling of Retained Proteins and PP2b

After the filtration experiment, the filter chamber was opened and the **PP2b** supramolecular layer was scratched off the CA support and suspended in MOPS buffer solution (1.5 ml). It was vortexed for 1 h in order to disentangle the **PP2b** supramolecular fibers, thus releasing retained proteins (R). In order to separate **PP2b** from the retained proteins, the solution was centrifuged for 15 minutes at 20800 g (using a desktop centrifuge). The supernatant contained retained proteins while the pellet (P) contained **PP2b** together with some retained proteins that could not be extracted. Both supernatant and pellet were analyzed separately by SDS-PAGE (Figure S6 in Text S1), whereby the pellet was highly concentrated due to its small volume. In order to purify **PP2b** in the pellet from residual proteins and other organic and inorganic contaminations, ten such pellets were dissolved in 20 ml of a water/ethanol mixture (2∶3, v/v). Subsequently, **PP2b** was extracted with dichloromethane (40 ml). The colorless aqueous phase was discarded. The organic phase was dried in high vacuum. The solid was washed with hexane (3×50 ml), and with water (1×50 ml). Subsequently, it was purified by silica column chromatography, eluting successively with: 1) 200 ml CHCl_3_, 2) 200 ml CHCl_3_/MeOH (98∶2, v/v), 3) 200 ml CHCl_3_/MeOH (92∶8, v/v). The product contained pure **PP2b**, as revealed by ^1^H-NMR (Figure S2 in Text S1). Nearly quantitative recycling was achieved.

## Supporting Information

Text S1 **Further experimental procedures and specifications, Supporting Figures S1–S16, and Supporting Tables S1–S5.**
(DOC)

## References

[1] Rybtchinski B (2011) Adaptive Supramolecular Nanomaterials Based on Strong Noncovalent Interactions. ACS Nano 5: 6791–6818. 21870803 10.1021/nn2025397

[2] Aida T, Meijer EW, Stupp SI (2012) Functional Supramolecular Polymers. Science 335: 813–817. 22344437 10.1126/science.1205962 PMC3291483

[3] Krieg E, Rybtchinski B (2011) Noncovalent Water-Based Materials: Robust yet Adaptive. Chem Eur J 17: 9016–9026. 21726009 10.1002/chem.201100809

[4] Stupp SI, LeBonheur V, Walker K, Li LS, Huggins KE, et al (1997) Supramolecular Materials: Self-Organized Nanostructures. Science 276: 384–389. 9103190 10.1126/science.276.5311.384

[5] Cordier P, Tournilhac F, Soulie-Ziakovic C, Leibler L (2008) Self-healing and thermoreversible rubber from supramolecular assembly. Nature 451: 977–980. 18288191 10.1038/nature06669

[6] Lehn JM (1993) Supramolecular chemistry. Science 260: 1762–1763. 8511582 10.1126/science.8511582

[7] Whitesides GM, Mathias JP, Seto CT (1991) Molecular self-assembly and nanochemistry: a chemical strategy for the synthesis of nanostructures. Science 254: 1312–1319. 1962191 10.1126/science.1962191

[8] Stoddart JF (2009) Thither supramolecular chemistry? Nature Chem 1: 14–15. 21378786 10.1038/nchem.142

[9] Wang Q, Mynar JL, Yoshida M, Lee E, Lee M, et al (2010) High-water-content mouldable hydrogels by mixing clay and a dendritic molecular binder. Nature 463: 339–343. 20090750 10.1038/nature08693

[10] Fenske T, Korth H-G, Mohr A, Schmuck C (2012) Advances in Switchable Supramolecular Nanoassemblies. Chem Eur J 18: 738–755. 22170585 10.1002/chem.201102435

[11] Sijbesma RP, Beijer FH, Brunsveld L, Folmer BJB, Hirschberg JHKK, et al (1997) Reversible polymers formed from self-complementary monomers using quadruple hydrogen bonding. Science 278: 1601–1604. 9374454 10.1126/science.278.5343.1601

[12] Capito RM, Azevedo HS, Velichko YS, Mata A, Stupp SI (2008) Self-assembly of large and small molecules into hierarchically ordered sacs and membranes. Science 319: 1812–1816. 18369143 10.1126/science.1154586

[13] Zhang S (2002) Emerging biological materials through molecular self-assembly. Biotechnology Advances 20: 321–339. 14550019 10.1016/s0734-9750(02)00026-5

[14] Dankers PYW, Meijer EW (2007) Supramolecular Biomaterials. A Modular Approach towards Tissue Engineering. Bull Chem Soc Jpn 80: 2047–2073.

[15] Stupp SI (2010) Self-Assembly and Biomaterials. Nano Lett 10: 4783–4786. 21028843 10.1021/nl103567y PMC3175009

[16] Lim Y-b, Moon K-S, Lee M (2008) Rod–coil block molecules: their aqueous self-assembly and biomaterials applications. J Mater Chem 18: 2909–2909.

[17] Percec V, Wilson DA, Leowanawat P, Wilson CJ, Hughes AD, et al (2010) Self-Assembly of Janus Dendrimers into Uniform Dendrimersomes and Other Complex Architectures. Science 328: 1009–1014. 20489021 10.1126/science.1185547

[18] Huang Z, Lee H, Lee E, Kang S-K, Nam J-M, et al (2011) Responsive nematic gels from the self-assembly of aqueous nanofibres. Nat Commun 2: 459–459. 21897370 10.1038/ncomms1465

[19] Shah RN, Shah Na, Del Rosario Lim MM, Hsieh C, Nuber G, et al (2010) Supramolecular design of self-assembling nanofibers for cartilage regeneration. PNAS 107: 3293–3298. 20133666 10.1073/pnas.0906501107 PMC2840471

[20] Saxena A, Tripathi BP, Kumar M, Shahi VK (2009) Membrane-based techniques for the separation and purification of proteins: An overview. Adv Coll Int Sci 145: 1–22. 10.1016/j.cis.2008.07.004 18774120

[21] van Reis R, Zydney A (2001) Membrane separations in biotechnology. Curr Opin Biotechnol 12: 208–211. 11287239 10.1016/s0958-1669(00)00201-9

[22] van Reis R, Zydney A (2007) Bioprocess membrane technology. J Membr Sci 297: 16–50.

[23] Lightfoot EN, Moscariello JS (2004) Bioseparations. Biotechnol Bioeng 87: 259–273. 15281101 10.1002/bit.20111

[24] Keller K, Friedmann T, Boxman A (2001) The bioseparation needs for tomorrow. Trends Biotechnol 19: 438–441. 11602307 10.1016/s0167-7799(01)01803-0

[25] Bhattacharjee S, Bhattacharjee C, Datta S (2006) Studies on the fractionation of beta-lactoglobulin from casein whey using ultrafiltration and ion-exchange membrane chromatography. J Membr Sci 275: 141–150.

[26] Lu J, Wan Y, Cui Z (2005) Fractionation of Lysozyme and Chicken Egg Albumin Using Ultrafiltration with 30-kDa Commercial Membranes. Ind Eng Chem Res 44: 7610–7616.

[27] Peeva PD, Knoche T, Pieper T, Ulbricht M (2012) Performance of Thin-Layer Hydrogel Polyethersulfone Composite Membranes during Dead-End Ultrafiltration of Various Protein Solutions. Ind Eng Chem Res 51: 7231–7241.

[28] Illanes As (2008) Enzyme biocatalysis: principles and applications: Springer.

[29] Ulbricht M (2006) Advanced functional polymer membranes. Polymer 47: 2217–2262.

[30] Vandezande P, Gevers LEM, Vankelecom IFJ (2008) Solvent resistant nanofiltration: separating on a molecular level. Chem Soc Rev 37: 365–405. 18197351 10.1039/b610848m

[31] Benfer S, Árki P, Tomandl G (2004) Ceramic membranes for filtration applications - preparation and characterization. Adv Eng Mater 6: 495–500.

[32] Tokarev I, Minko S (2009) Multiresponsive, Hierarchically Structured Membranes: New, Challenging, Biomimetic Materials for Biosensors, Controlled Release, Biochemical Gates, and Nanoreactors. Adv Mater 21: 241–247.

[33] Yang Q, Adrus N, Tomicki F, Ulbricht M (2011) Composites of functional polymeric hydrogels and porous membranes. J Mater Chem 21: 2783–2811.

[34] Beginn U (1998) Supramolecular templates as porogenes. Adv Mater 10: 1391–1394.

[35] Yang SY, Yang J-A, Kim E-S, Jeon G, Oh EJ, et al (2010) Single-File Diffusion of Protein Drugs through Cylindrical Nanochannels. ACS Nano 4: 3817–3822. 20507175 10.1021/nn100464u

[36] Phillip WA, O’Neill B, Rodwogin M, Hillmyer MA, Cussler EL (2010) Self-Assembled Block Copolymer Thin Films as Water Filtration Membranes. ACS Appl Mater Inter 2: 847–853. 10.1021/am900882t 20356290

[37] Beginn U, Zipp G, Möller M (2000) Functional Membranes Containing Ion-Selective Matrix-Fixed Supramolecular Channels. Adv Mater 12: 510–513.

[38] Krieg E, Weissman H, Shirman E, Shimoni E, Rybtchinski B (2011) A recyclable supramolecular membrane for size-selective separation of nanoparticles. Nature Nanotech 6: 141–146. 10.1038/nnano.2010.274 21258332

[39] Lu Y, Suzuki T, Zhang W, Moore JS, Marinas BJ (2007) Nanofiltration membranes based on rigid star amphiphiles. Chem Mater 19: 3194–3204. 10.1021/es070157s 17937310

[40] Peinemann K-V, Abetz V, Simon PFW (2007) Asymmetric superstructure formed in a block copolymer via phase separation. Nat Mater 6: 992–996. 17982467 10.1038/nmat2038

[41] Nunes SP, Behzad AR, Hooghan B, Sougrat R, Karunakaran M, et al (2011) Switchable pH-Responsive Polymeric Membranes Prepared via Block Copolymer Micelle Assembly. ACS Nano 5: 3516–3522. 21504167 10.1021/nn200484v

[42] Tyagi P, Deratani A, Bouyer D, Cot D, Gence V, et al (2012) Dynamic Interactive Membranes with Pressure-Driven Tunable Porosity and Self-Healing Ability. Angew Chem Int Ed 51: 7166–7170. 10.1002/anie.201201686 22696305

[43] Dankers PYW, Boomker JM, Huizinga-van der Vlag A, Smedts FMM, Harmsen MC, et al (2010) The use of fibrous, supramolecular membranes and human tubular cells for renal epithelial tissue engineering: towards a suitable membrane for a bioartificial kidney. Macromol Biosci 10: 1345–1354. 20715132 10.1002/mabi.201000146

[44] Uehara H, Kakiage M, Sekiya M, Sakuma D, Yamonobe T, et al (2009) Size-Selective Diffusion in Nanoporous but Flexible Membranes for Glucose Sensors. ACS Nano 3: 924–932. 19323485 10.1021/nn8008728

[45] Ball P (2008) Water as an active constituent in cell biology. Chem Rev 108: 74–108. 18095715 10.1021/cr068037a

[46] Blokzijl W, Engberts JBFN (1993) Hydrophobic effects. Opinions and facts. Angew Chem Int Ed 32: 1545–1579.

[47] Chandler D (2005) Interfaces and the driving force of hydrophobic assembly. Nature 437: 640–647. 16193038 10.1038/nature04162

[48] Oshovsky GV, Reinhoudt DN, Verboom W (2007) Supramolecular Chemistry in Water. Angew Chem Int Ed 46: 2366–2393. 10.1002/anie.200602815 17370285

[49] Ryu J-H, Hong D-J, Lee M (2008) Aqueous self-assembly of aromatic rod building blocks. Chem Commun: 1043–1054. 10.1039/b713737k 18292887

[50] Görl D, Zhang X, Würthner F (2012) Molecular Assemblies of Perylene Bisimide Dyes in Water. Angew Chem Int Ed 51: 6328–6348. 10.1002/anie.201108690 22573415

[51] Krieg E, Shirman E, Weissman H, Shimoni E, Wolf SG, et al (2009) Supramolecular gel based on a perylene diimide dye: multiple stimuli responsiveness, robustness, and photofunction. J Am Chem Soc 131: 14365–14373. 19807182 10.1021/ja903938g

[52] Würthner F (2004) Perylene bisimide dyes as versatile building blocks for functional supramolecular architectures. Chem Commun: 1564–1579. 10.1039/b401630k 15263926

[53] Lo Nostro P, Ninham BW, Milani S, Lo Nostro A, Pesavento G, et al (2006) Hofmeister effects in supramolecular and biological systems. Biophys Chem 124: 208–213. 16678961 10.1016/j.bpc.2006.04.004

[54] Gibb BC (2011) Supramolecular Assembly and Binding in Aqueous Solution: Useful Tips Regarding the Hofmeister and Hydrophobic Effects. Isr J Chem 51: 798–806.

[55] Corbin PS, Zimmerman SC (1998) Self-Association without Regard to Prototropy. A Heterocycle That Forms Extremely Stable Quadruply Hydrogen-Bonded Dimers. J Am Chem Soc 120: 9710–9711.

[56] Schmuck C, Wienand W (2003) Highly Stable Self-Assembly in Water: Ion Pair Driven Dimerization of a Guanidiniocarbonyl Pyrrole Carboxylate Zwitterion. J Am Chem Soc 125: 452–459. 12517158 10.1021/ja028485+

[57] Chen Z, Lohr A, Saha-Möller CR, Würthner F (2009) Self-assembled pi-stacks of functional dyes in solution: structural and thermodynamic features. Chem Soc Rev 38: 564–584. 19169466 10.1039/b809359h

[58] Prins LJ, Reinhoudt DN, Timmerman P (2001) Noncovalent Synthesis Using Hydrogen Bonding. Angew Chem Int Ed 40: 2382–2426. 10.1002/1521-3773(20010702)40:13<2382::aid-anie2382>3.0.co;2-g 11443654

[59] Tidhar Y, Weissman H, Wolf SG, Gulino A, Rybtchinski B (2011) Pathway-Dependent Self-Assembly of Perylene Diimide/Peptide Conjugates in Aqueous Medium. Chem Eur J 17: 6068–6075. 21542033 10.1002/chem.201003419

[60] Kane RS, Deschatelets P, Whitesides GM (2003) Kosmotropes Form the Basis of Protein-Resistant Surfaces. Langmuir 19: 2388–2391.

[61] Kingshott P, Thissen H, Griesser HJ (2002) Effects of cloud-point grafting, chain length, and density of PEG layers on competitive adsorption of ocular proteins. Biomaterials 23: 2043–2056. 11996046 10.1016/s0142-9612(01)00334-9

[62] Dalsin JL, Lin L, Tosatti S, Vörös J, Textor M, et al (2004) Protein Resistance of Titanium Oxide Surfaces Modified by Biologically Inspired mPEG-DOPA. Langmuir 21: 640–646. 10.1021/la048626g 15641834

[63] Brady D, Jordaan J (2009) Advances in enzyme immobilisation. Biotechnol Lett 31: 1639–1650. 19590826 10.1007/s10529-009-0076-4

[64] Sheldon RA (2007) Enzyme Immobilization: The Quest for Optimum Performance. Adv Synth Catal 349: 1289–1307.

[65] Spahn C, Minteer SD (2008) Enzyme Immobilization in Biotechnology. Recent Pat Eng 2: 195–200.

[66] Tran DN, Balkus KJ (2011) Perspective of Recent Progress in Immobilization of Enzymes. ACS Catal 1: 956–968.

[67] see for example technical specifications of Koch Membrane Systems HFM-180 or Sartorius stedim VF20P4, VF20C4.

[68] Röthlisberger D, Khersonsky O, Wollacott AM, Jiang L, DeChancie J, et al (2008) Kemp elimination catalysts by computational enzyme design. Nature 453: 190–195. 18354394 10.1038/nature06879

[69] Khersonsky O, Röthlisberger D, Wollacott AM, Murphy P, Dym O, et al (2011) Optimization of the in-silico-designed kemp eliminase KE70 by computational design and directed evolution. J Mol Biol 407: 391–412. 21277311 10.1016/j.jmb.2011.01.041 PMC3889864

[70] Maurus R, Nguyen NT, Stokell DJ, Ayed A, Hultin PG, et al (2003) Insights into the Evolution of Allosteric Properties. The NADH Binding Site of Hexameric Type II Citrate Synthases. Biochemistry 42: 5555–5565. 12741811 10.1021/bi020622s

[71] Nguyen NT, Maurus R, Stokell DJ, Ayed A, Duckworth HW, et al (2001) Comparative Analysis of Folding and Substrate Binding Sites between Regulated Hexameric Type II Citrate Synthases and Unregulated Dimeric Type I Enzymes. Biochemistry 40: 13177–13187. 11683626 10.1021/bi010408o

[72] Joerger AC, Gosse C, Fessner W-D, Schulz GE (2000) Catalytic Action of Fuculose 1-Phosphate Aldolase (Class II) As Derived from Structure-Directed Mutagenesis. Biochemistry 39: 6033–6041. 10821675 10.1021/bi9927686

[73] Baker R (2004) Membrane Technology and Applications. Menlo Park: Wiley.

[74] Srere PA, Brazil H, Gonen L (1963) The Citrate Condensing Enzyme of Pigeon Breast Muscle and Moth Flight Muscle. Acta Chem Scand 17: S129–S134.

[75] Bradford MM (1976) A rapid and sensitive method for the quantitation of microgram quantities of protein utilizing the principle of protein-dye binding. Anal Biochem 72: 248–254. 942051 10.1016/0003-2697(76)90527-3

[76] Miller JH (1972) Experiments in molecular genetics. New York: Cold Spring Harbor Laboratory.

[77] Cao L, van Rantwijk F, Sheldon RA (2000) Cross-Linked Enzyme Aggregates: A Simple and Effective Method for the Immobilization of Penicillin Acylase. Org Lett 2: 1361–1364. 10814447 10.1021/ol005593x

[78] Torres-Salas P, del Monte-Martinez A, Cutiño-Avila B, Rodriguez-Colinas B, Alcalde M, et al (2011) Immobilized Biocatalysts: Novel Approaches and Tools for Binding Enzymes to Supports. Adv Mater 23: 5275–5282. 22299142 10.1002/adma.201101821

[79] Onda M, Lvov Y, Ariga K, Kunitake T (1996) Sequential reaction and product separation on molecular films of glucoamylase and glucose oxidase assembled on an ultrafilter. J Ferment Bioeng 82: 502–506.

[80] Sayle RA, Milner-White EJ (1995) RASMOL: biomolecular graphics for all. Trends Biochem Sci 20: 374–374. 7482707 10.1016/s0968-0004(00)89080-5

[81] Bernstein HJ (2000) Recent changes to RasMol, recombining the variants. Trends Biochem Sci 25: 453–455. 10973060 10.1016/s0968-0004(00)01606-6

[82] Serp D, Mueller M, Stockar Uv, Marison IW (2002) Low-temperature electron microscopy for the study of polysaccharide ultrastructures in hydrogels. I. Theoretical and technical considerations. Biotechnol Bioeng 79: 243–252. 12115412 10.1002/bit.10286

[83] Serp D, Mueller M, Stockar Uv, Marison IW (2002) Low-temperature electron microscopy for the study of polysaccharide ultrastructures in hydrogels. II. Effect of temperature on the structure of Ca^2+^-alginate beads. Biotechnol Bioeng 79: 253–259. 12115413 10.1002/bit.10287

[84] Matzelle T, Reichelt R (2008) Review: Hydro-, micro- and nanogels studied by complementary measurements based on SEM and SFM. Acta Microscopica 17: 45–61.

[85] Menger FM, Seredyuk VA, Apkarian RP, Wright ER (2002) Colloidal Assemblies of Branched Geminis Studied by Cryo-etch-HRSEM. J Am Chem Soc 124: 12408–12409. 12381171 10.1021/ja021025w

