# Supplementary material for: Separation, Immobilization, and Biocatalytic Utilization of Proteins by a Supramolecular Membrane
Source: PLoS One. 2013 May 10;8(5):e63188. doi: 10.1371/journal.pone.0063188 (PMC3651134; doi:10.1371/journal.pone.0063188)
Supplement: Text S1 — Further experimental procedures and specifications, Supporting Figures S1–S16, and Supporting Tables S1–S5. (DOC) [file pone.0063188.s001.doc]

**Supporting Text S1**

**Separation, immobilization, and biocatalytic utilization of proteins by a supramolecular membrane**

Elisha Krieg, Shira Albeck, Haim Weissman, Eyal Shimoni, and Boris Rybtchinski*

**Contents**

[S1. Protein Specifications 2](#__RefHeading___Toc339376735)

[S2. Buffer Solutions 2](#__RefHeading___Toc339376736)

[S3. MALDI-TOF 3](#__RefHeading___Toc339376737)

[S4. NMR 4](#__RefHeading___Toc339376738)

[S5. Gel Filtration Chromatography (GFC) 5](#__RefHeading___Toc339376739)

[S6. Dynamic Light Scattering (DLS) 6](#__RefHeading___Toc339376740)

[S7. SDS-PAGEs 6](#__RefHeading___Toc339376741)

[S8. UV/Vis Spectra 8](#__RefHeading___Toc339376742)

[S9. Protein Separation over a Membrane Prepared from Recycled PP2b 9](#__RefHeading___Toc339376743)

[S10. Activity of Filtered KE70 10](#__RefHeading___Toc339376744)

[S11. Activity of Recycled CS 11](#__RefHeading___Toc339376745)

[S12. CS Immobilization and Biocatalysis 12](#__RefHeading___Toc339376746)

[S13. Leaching of CS 13](#__RefHeading___Toc339376747)

[S14. β-Gal Immobilization and Biocatalysis 14](#__RefHeading___Toc339376748)

[S15. Leaching of β-Gal 16](#__RefHeading___Toc339376749)

[S16. References 17](#__RefHeading___Toc339376750)

# Protein specifications

The following proteins were produced by the Israel Structural Proteomics Center (ISPC)**;** *EIIBCA* - N-terminal domain of EIIBCA-Bgl (residues 2-84) – (UniProtKB;P08722), *KE70* - In silico designed Kemp eliminase (PDB: 3Q2D), *CS* - Citrate synthase 1- (UniProtKB; Q10530). *LCD* - L-carnitine dehydratase/bile acid-inducible protein F- (UniProtKB;A6W2K8) was the gift of U. Alcolombri and Prof. D. Tawfik (The Weizmann Institute). *BSA (monomeric)* was the gift of Amona Ali and Zeev Gross (Technion). The following proteins are commercial**;** *Aldolase –* L-fuculose-1-phosphate aldolase (Amersham Biosciences, catalog no.:17-0441-01), *BSA (oligomeric)* - Bovine serum albumin, molecular biology grade, protease free, fraction V starting material (New England Biolabs, catalog no.: B9001S), *β-Gal* - β-D-Galactoside galactohydrolase lactase (Sigma-Aldrich, catalog no.: G6008, Lot:109K8618V)

# Buffer solutions

MOPS buffer solution (pH 7.0)

209 mg 3-(N-Morpholino)propanesulfonic acid (**MOPS**; 1 mmol; final concentration: 20 mM), 261 mg KCl (3.5 mmol; final concentration: 70 mM), and 47.6 mg MgCl_2_ (0.5 mmol; final concentration: 10 mM) were dissolved in water (50 ml). The solution was adjusted with NaOH (1M) to pH 7.0 and stored at r.t..

Z-buffer solution (pH 7.0)[1]

570 mg Na_3_PO_4_ · 12 H_2_O (1.5 mmol; final concentration: 30 mM), 546 mg NaH_2_PO_4_ · 2 H_2_O (3.5 mmol; final concentration: 70 mM), 37.3 mg KCl (0.5 mmol; final concentration: 10 mM), and 12.5 mg MgSO_4_ · 7 H_2_O (0.05 mmol; final concentration: 1 mM) were dissolved in water (50 ml). The solution was adjusted with NaOH (1M) to pH 7.0 and stored at 4°C. On the day of its use, 140 μl β-mercaptoethanol was added.

HEPES buffer solution (pH 7.5)

1.46 g NaCl (25 mmol; final concentration: 100 mM) was dissolved in water (237.5 ml) and 12.5 ml of a stock solution of 4-(2-hydroxyethyl)-1-piperazineethanesulfonic acid (**HEPES**, 1M; final concentration: 50 mM) was added. The solution was adjusted to pH 7.5 and stored at r.t..

# MALDI-TOF

**A B**


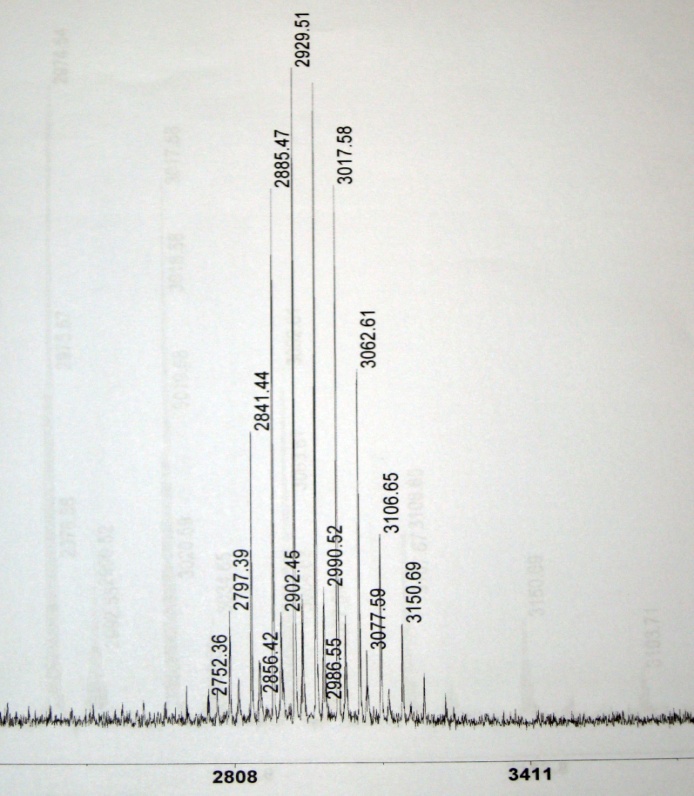

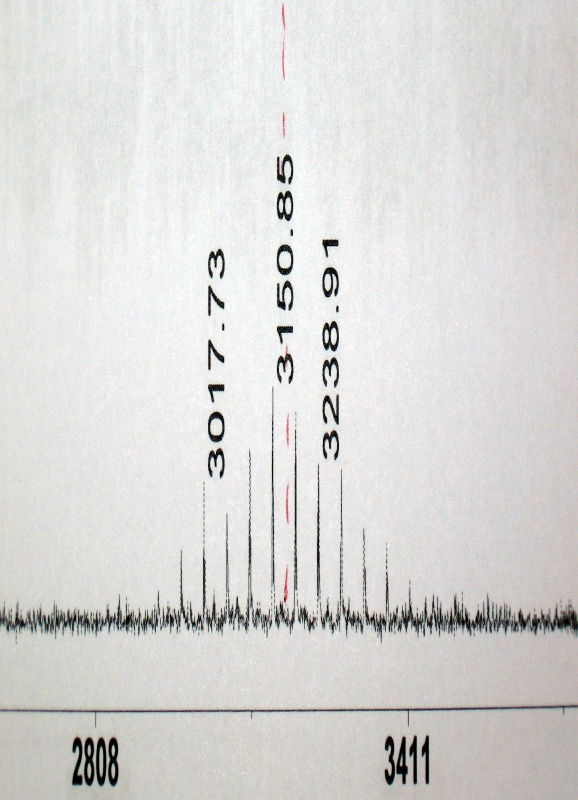


Figure S1. Molecular weight of the membrane material. MALDI-TOF mass spectra of (A) PP2b(s). (B) PP2b(l).

# NMR

**Figure S2. Purity of PP2b before and after recycling.** ^1^H-NMR spectra of **PP2b** before separation of proteins (top), and **PP2b** recycled after protein filtration (bottom).

# Gel filtration chromatography (GFC)

**
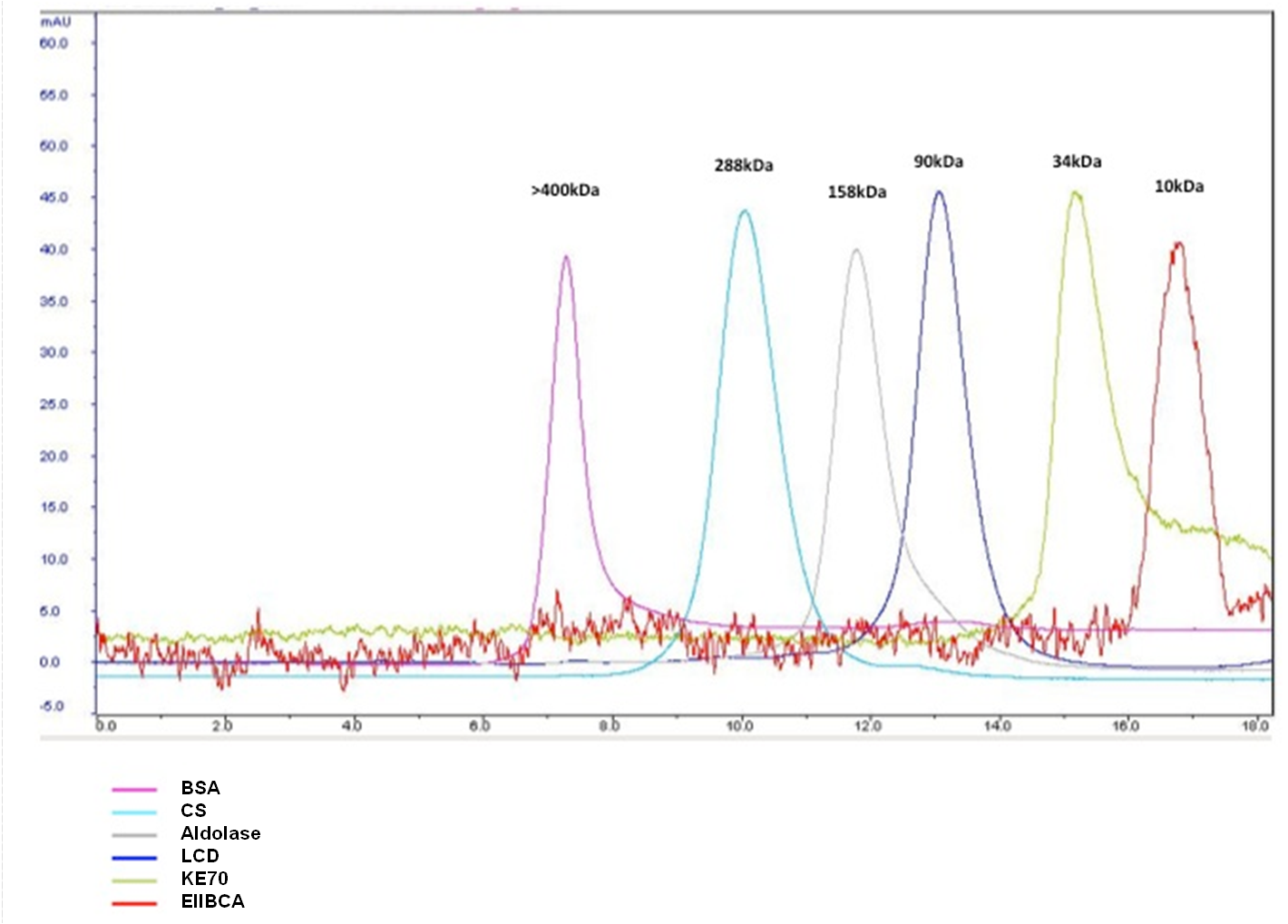
**

**Figure S3. Molecular weight of various proteins.** Gel filtration chromatograms of BSA (olig.), CS (hex.), Aldolase, LCD, KE70, and EIIBCA.

# Dynamic light scattering (DLS)

**
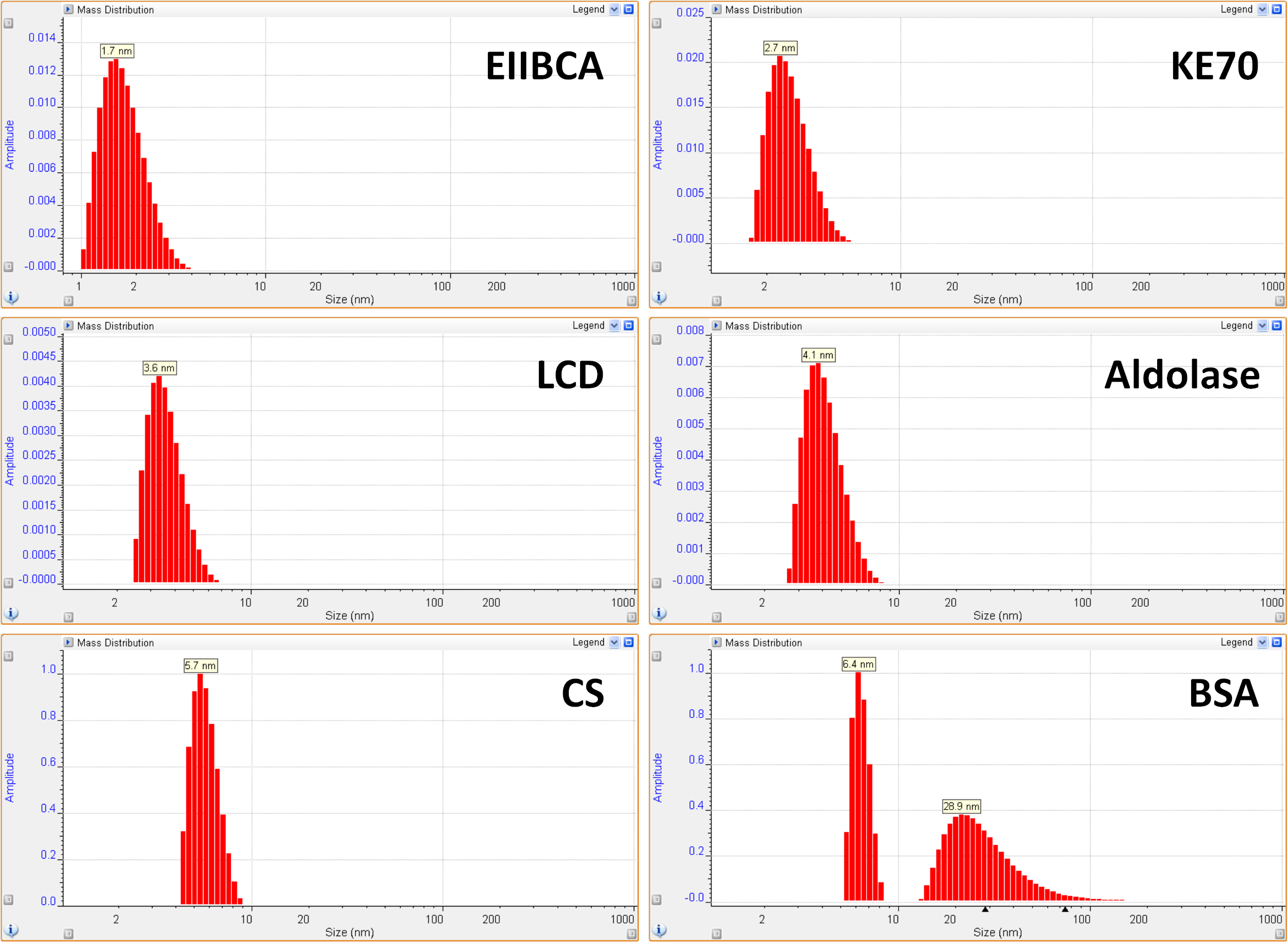
**

**Figure S4. Hydrodynamic radii of various proteins.** DLS size histograms of EIIBCA, KE70, LCD, Aldolase, CS, and BSA. All proteins were dissolved in MOPS buffer solution at a concentration of 0.3 mg/ml.

# SDS-PAGEs

**
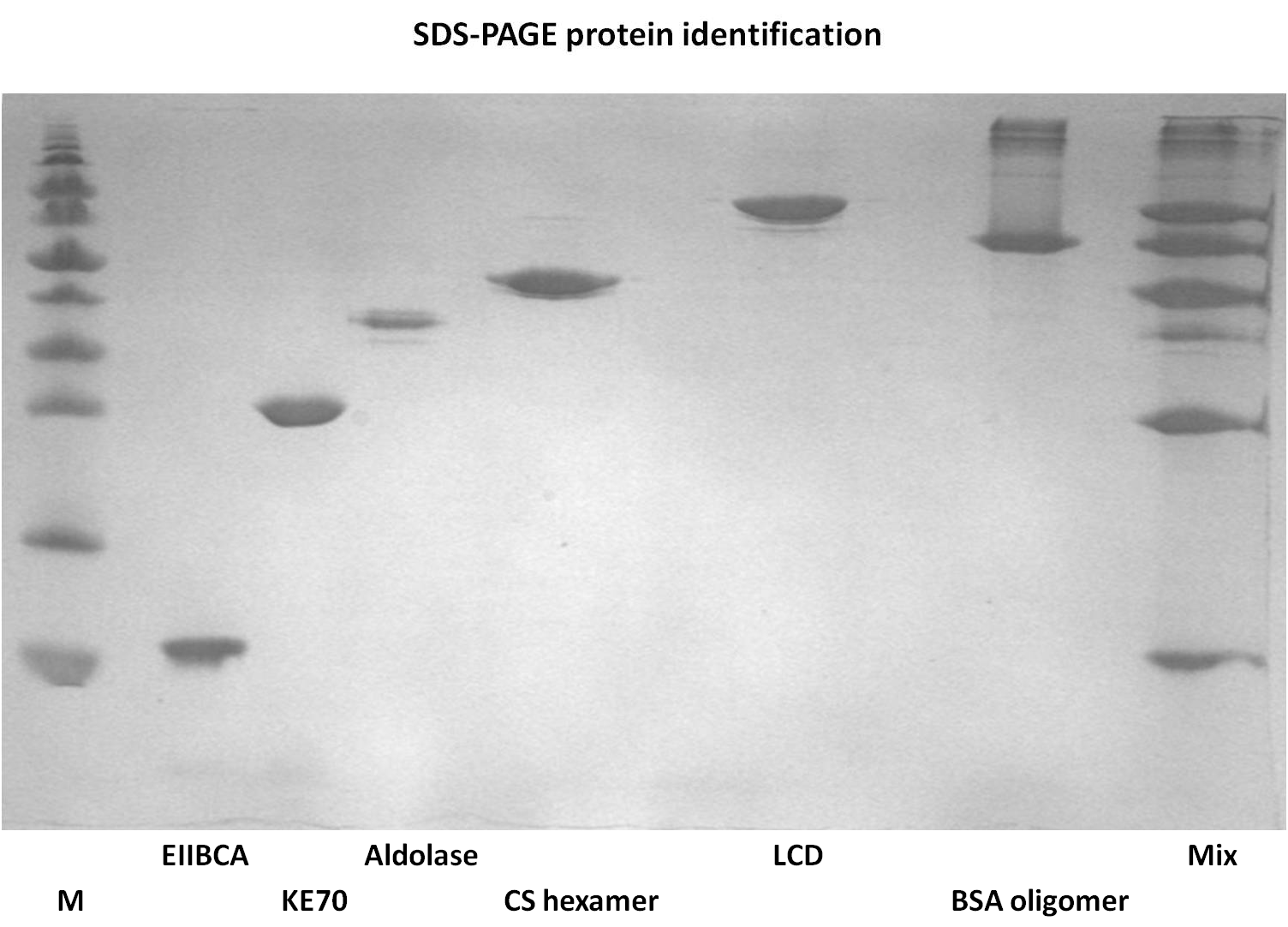
**

**Figure S5. SDS-PAGE of neat proteins in MOPS buffer solution.** BSA, CS, Aldolase, LCD, KE70, and EIIBCA, and their mixture. M = Molecular weight marker (170, 130, 95, 72, 55, 43, 34, 26, 17, 11 kDa).

**
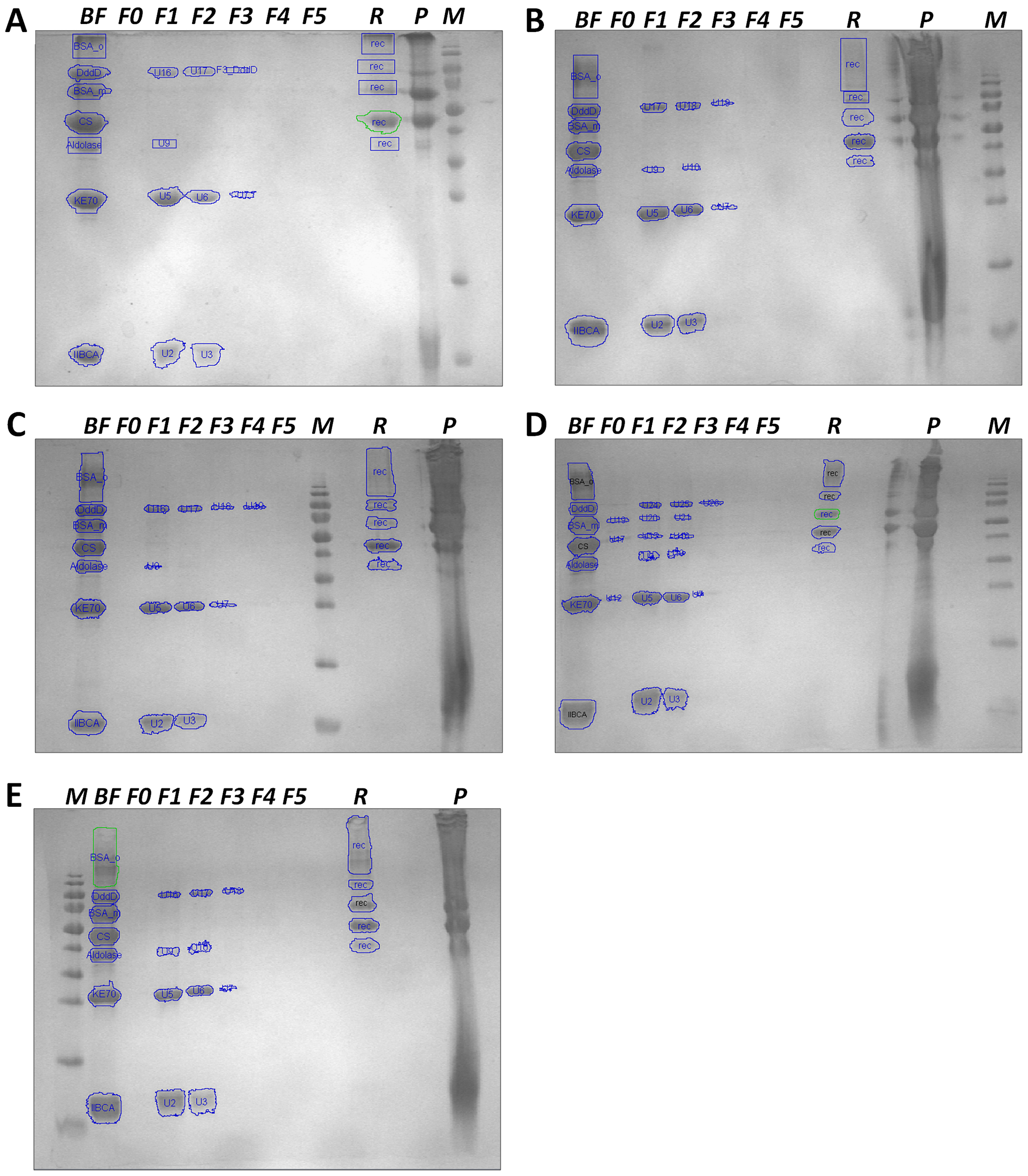
**

**Figure S6. SDS-PAGEs for five filtration experiments**. (A)-(E) BF = Before filtration, M = Molecular weight marker, R = Recycled, P = Pellet (highly concentrated). Selected areas for densitometric protein quantification are marked.

# UV/Vis spectra

**
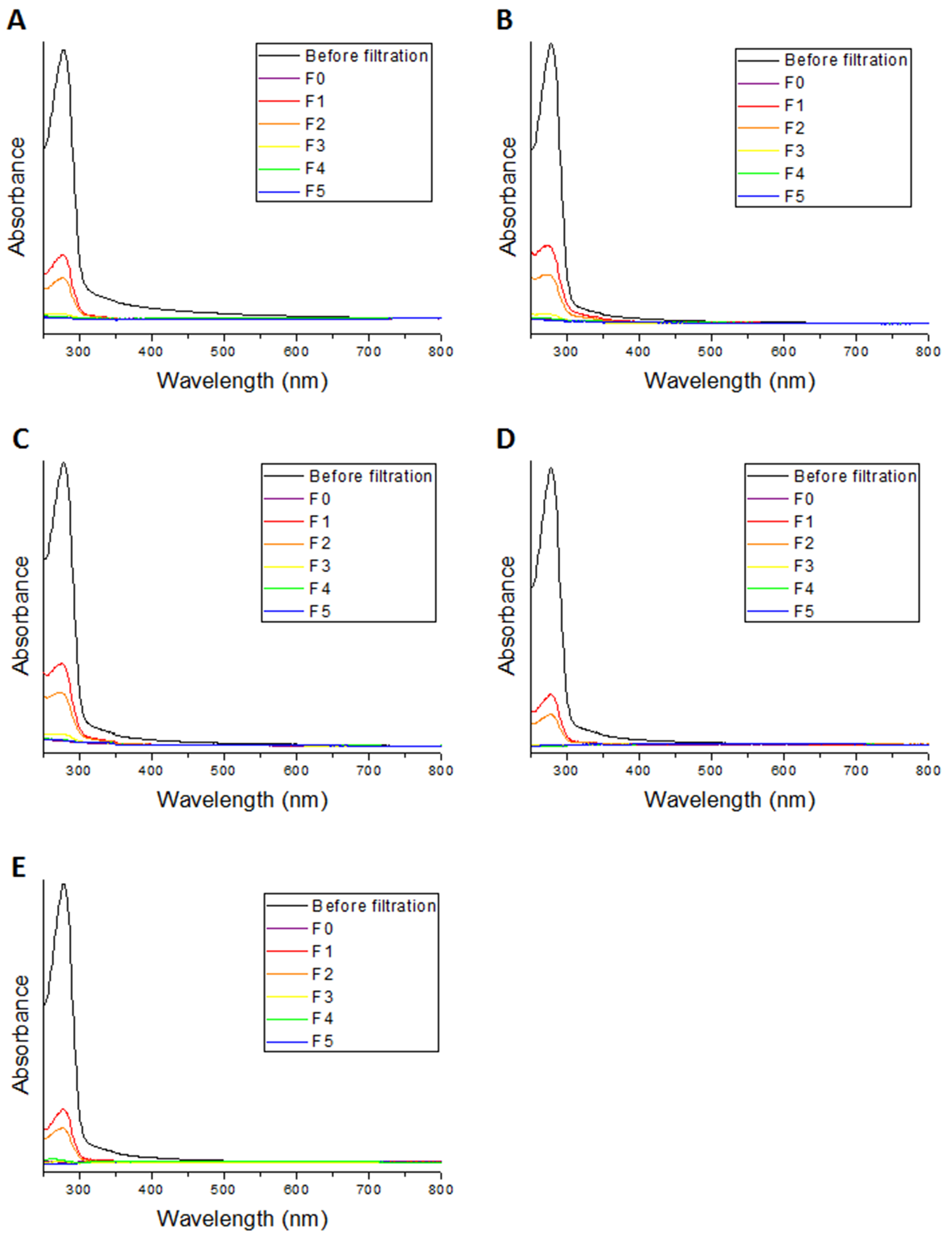
**

**Figure S7. Filtration of protein mixtures over the PP2b supramolecular membrane.** (A)-(E) UV/Vis spectra of protein mixtures before filtration and filtered fractions collected in 5 independent filtration experiments.

# Protein separation over a membrane prepared from recycled PP2b

**
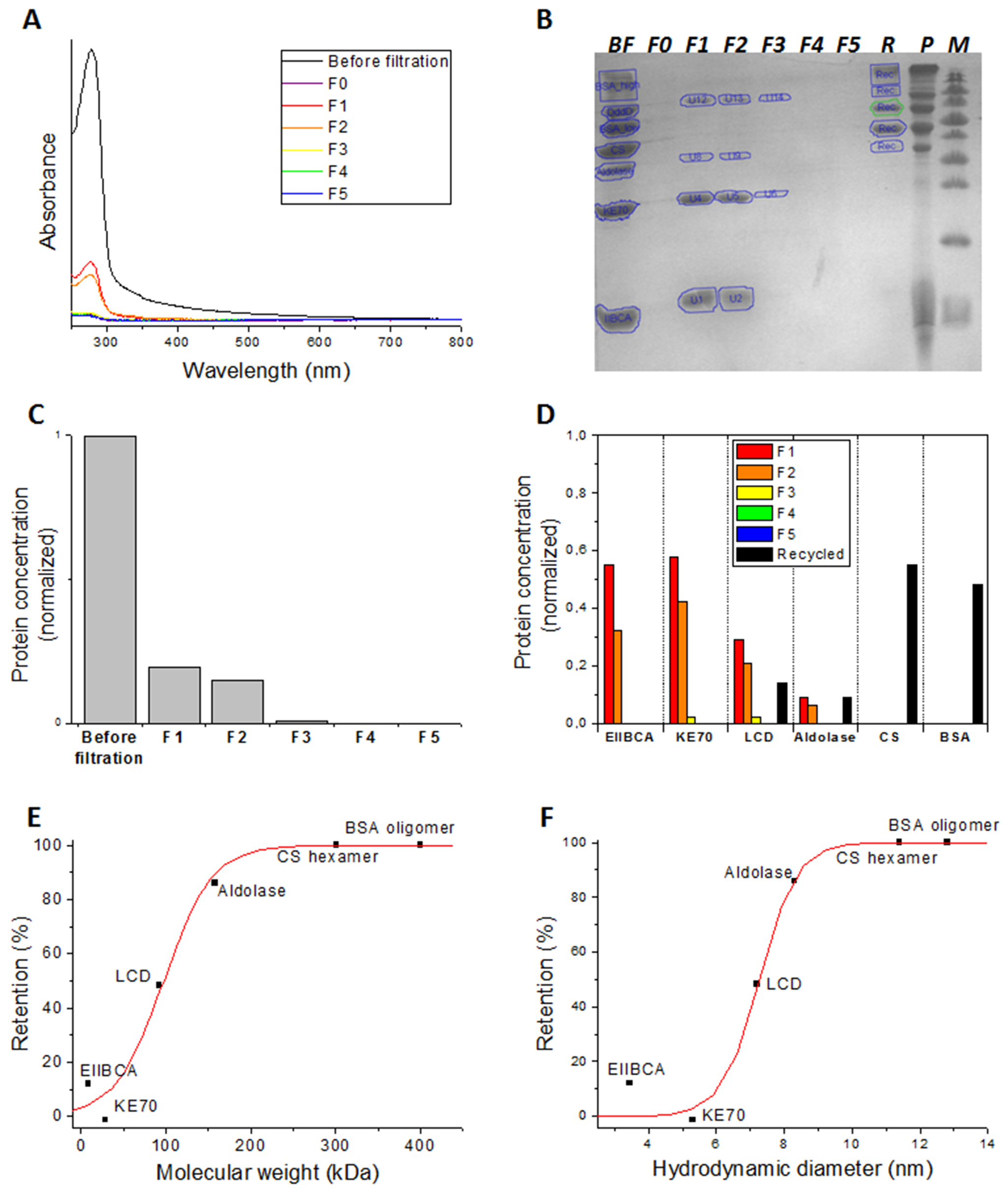
**

**Figure S8. Filtration of proteins over a supramolecular membrane fabricated from recycled** **PP2b**. (A) UV/Vis spectra of the protein mixture before filtration, and filtered fractions. (B) SDS-PAGE of the filtration experiment. BF = Before filtration, M = Molecular weight marker (170, 130, 95, 72, 55, 43, 34, 26, 17, 11 kDa), R = Recycled, P = Pellet (highly concentrated). Selected areas for densitometric protein quantification are marked. (C) Total protein concentration in the filtrate fractions F1-F5 as compared to the feed solution. (D) Protein concentrations (normalized with respect to the non-filtered solution) of fractions F1-F5, and recycled proteins. (E) Plot of protein retention against molecular weight (black data points) and sigmoid fit (red curve). (F) Plot of protein retention against hydrodynamic diameter (black data points) and sigmoid fit (red curve).

# Activity of filtered KE70

A solution of KE70 (4 ml, 0.3 mg/ml) in HEPES buffer was filtered over a freshly prepared supramolecular membrane. Due to some dilution in the filter chamber, the filtrate contained KE70 at 89.6% of its original concentration, based on absorbance at 280 nm (Figure S9, Table S1). The quantification of KE70 activity in the solution before filtration, in the filtrate, and in neat buffer solution (background reaction) was performed at 25°C by measuring the kinetics of the enzyme-catalyzed isomerisation of 5‑Nitrobezisoxazole via absorbance of the product at 380 nm (OD_380_).[2] For this, 15 μl of the test solution was diluted in 1.5 ml HEPES buffer in a UV/Vis cuvette, and OD_380_ was recorded for several minutes. Then the reaction was started by addition of 1 μl 5‑Nitrobezisoxazole from stock solution in acetonitrile (67 μM after dilution) and the kinetics measurement was continued for 10-15 minutes (Figure S10A). The slope of OD_380_ (ΔOD_380_ / Δt) in the linear region of the kinetics plot (Figure S10B) is proportional to the enzymatic activity under saturation condition. Thus, activity of the filtrate was determined to be 88.1% with respect to the original solution, whilst the concentration determined from OD at 280 nm was 89.6%. Therefore, taking into consideration dilution, the activity of filtered KE70 is ≥98%.


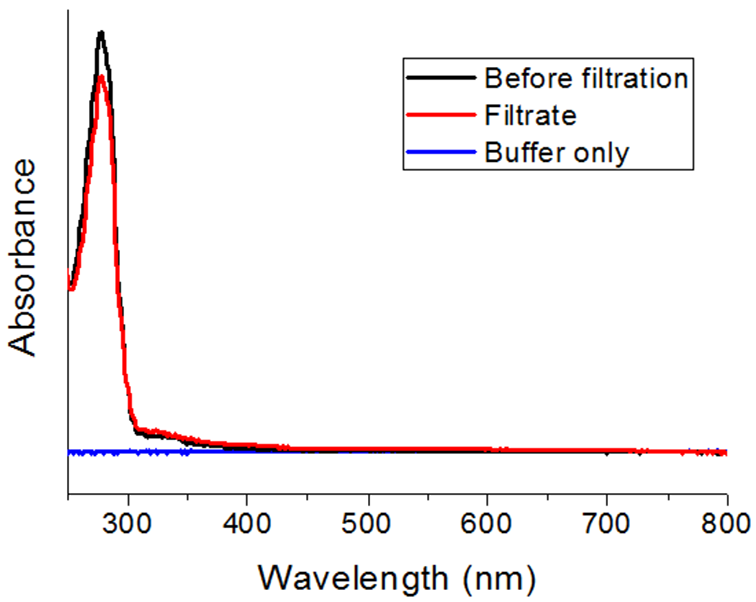


**Figure S9. UV/Vis spectra of KE70.** Absorption spectra of the solution before filtration, of the filtrate, and of the neat buffer solution.

**
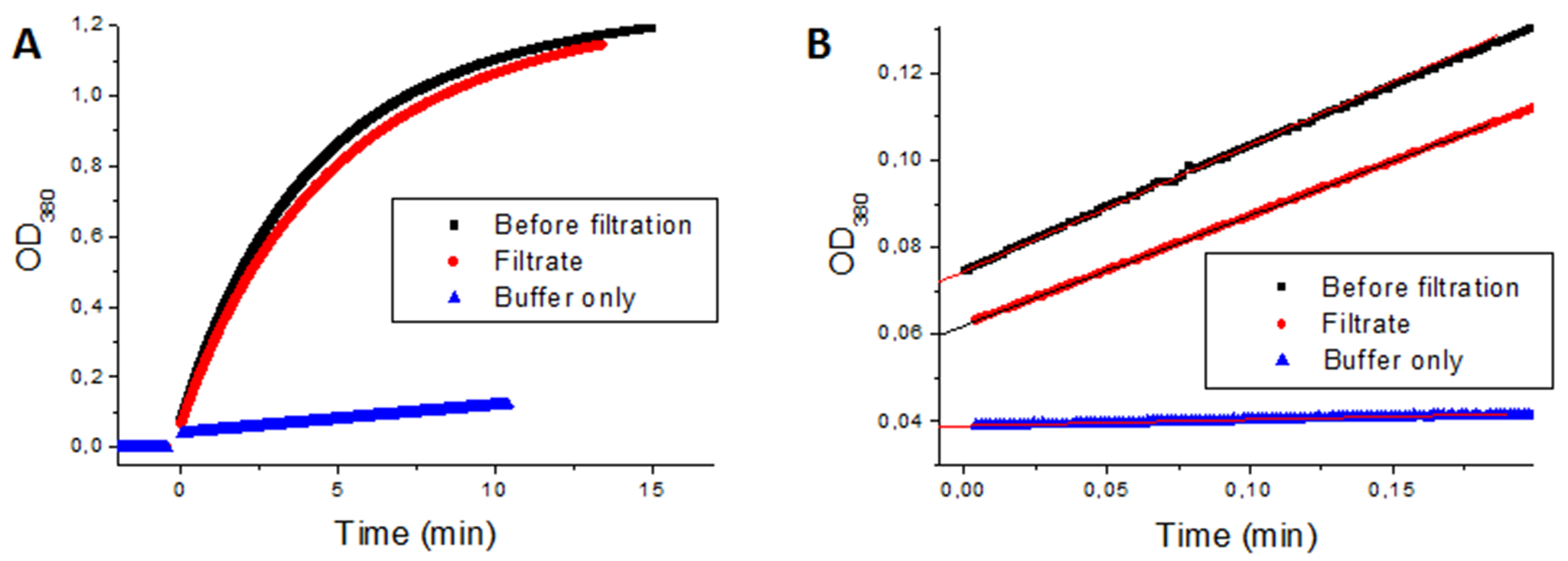
**

**Figure S10. Activity of filtered KE70.** Kinetics of the KE70 activity before filtration, after filtration, and of neat buffer solution, as revealed by the change in absorbance at 380 nm, following addition of 5-Nitrobezisoxazole (at t = 0 min). (A) Full experiment. (B) Linear range of enzyme kinetics and regression lines.

**Table S1.** Absorbance at 280 nm (OD_280_) for quantification of the relative enzyme concentrations, line slope of the linear range of the kinetics plot (ΔOD_380_ / Δt), relative KE70 concentration and activity.

|  | **OD_280_** |  | **ΔOD_380_ / Δt [min^-1^]** | **Relative Conc. [%]** | **Relative activity [%]** |
| --- | --- | --- | --- | --- | --- |
| **Before filtration** | 0.1123 |  | 0.2704 | 100 | 100 |
| **Filtrate** | 0.1007 |  | 0.2391 | 89.6 | 88.1 |
| **Buffer only** | 0.0003 |  | 0.0080 | 0 | 0 |

# Activity of recycled CS

CS (0.3 mg/ml) in HEPES buffer solution (1.5 ml) was filtered over a freshly prepared supramolecular membrane, followed by 6 ml neat buffer solution. The enzyme was recycled according to the regular procedure (see Experimental Section). Quantification of the protein concentration of recycled enzyme and of the enzyme solution before filtration was performed via Bradford test (Table S2).[3]

**Table S2.** Quantification of CS via Bradford test.

|  | **OD_600_** | **Conc. [mg/ml]** | **Relative Conc. [%]** |
| --- | --- | --- | --- |
| **Before filtration** | - | 0.30 | 100 |
| **Before filtration (10x diluted)** | 0.38 | 0.030 |  |
| **Recycled** | - | 0.079 | 26 |
| **Recycled (5x diluted)** | 0.20 | 0.016 |  |

The activity of CS before and after filtration was quantified at 25°C according to an activity assay based on a literature procedure[4]. In a UV/Vis cuvette, 143 μl of the enzyme-containing solution was diluted with HEPES buffers to a total volume of 1.43 ml. Then the following reagents were added successively: 6.0 μl of Ethylenediaminetetraacetic acid (**EDTA**; 500 mM; final concentration: 2 mM) in water, 15 μl of 5,5′-Dithiobis(2-nitrobenzoate) (**DTNB**; 10 mM; final concentration: 0.1 mM) in ethanol, and 15 μl of Acetly Coenzyme A (**Acetyl-CoA**; 14 mM; final concentration: 0.14 mM) in HEPES buffer. Absorbance at 412 nm (OD_412_) was recorded for a few minutes. Then 30 μl of Oxaloacetate (10 mM; final concentration: 0.2 mM) in HEPES buffer was added and OD_412_ was recorded for another 4-5 minutes (Figure S11A). The slope of OD_412_ (ΔOD_412_ / Δt) in the linear region of the kinetics plot (Figure S11B) is proportional to the enzymatic activity under saturation condition. Thus, activity of the recycled enzymes was determined to be **29%** with respect to the original solution (Table S3). This value is in good agreement with the concentration determined from Bradford test (26%), showing that enzymatic activity is conserved during the filtration and recycling process.


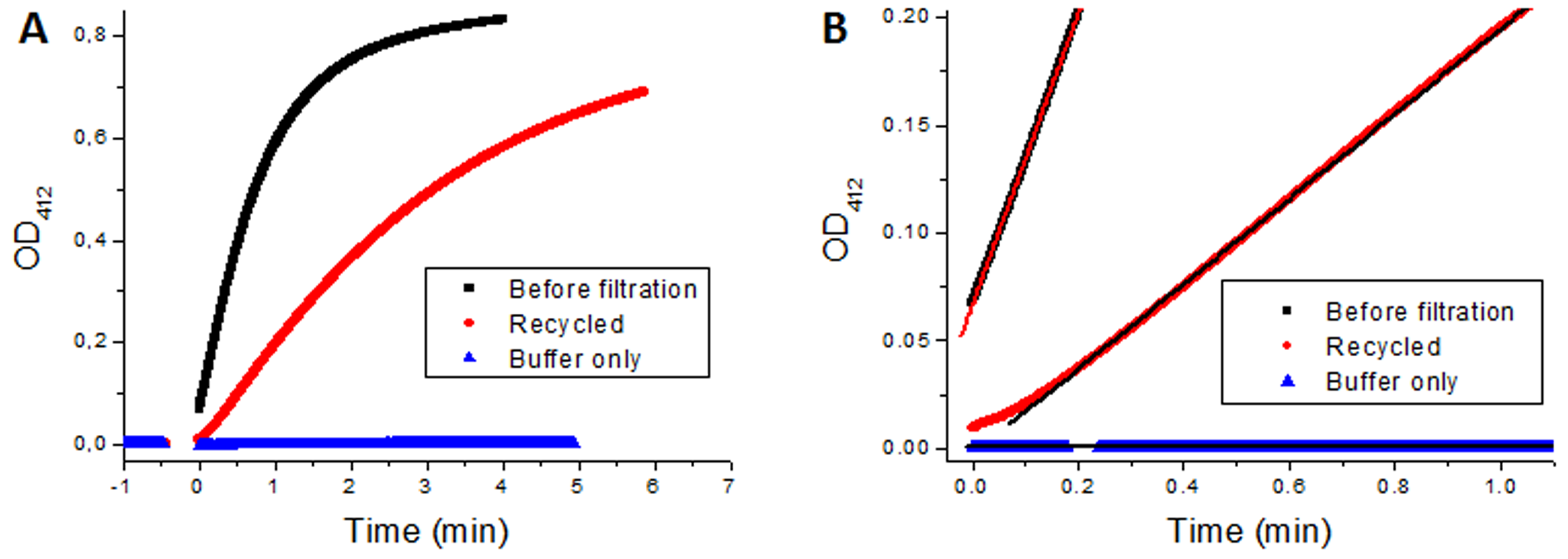


**Figure S11. Activity of recycled CS.** Kinetics of the CS activity before filtration, after filtration and recycling from the membrane, and of neat buffer solution, as revealed by the change in absorbance at 412 nm, following addition of Oxaloacetate (at t = 0 min). (A) Full experiment. (B) Linear range of enzyme kinetics and regression lines.

**Table S3**  Slope of the linear fits (ΔOD_412_ / Δt) in the initial CS activity kinetics, and calculated relative activity.

|  | **ΔOD_412_ / Δt [min^-1^]** | **Relative activity [%]** |
| --- | --- | --- |
| **Before filtration** | 0.6749 | 100 |
| **Recycled** | 0.1974 | 29 |
| **Buffer only** | 0.0004 | 0 |

# CS immobilization and biocatalysis

A solution of CS (2 ml, 0.3 mg/ml) in HEPES buffer was filtered over a freshly prepared supramolecular membrane. The membrane was rinsed with 10.5 ml of neat HEPES buffer to wash out traces of penetrating enzymes. The filtrate of the buffer solution was collected, in order to assess the amount of leaching enzymes (see section S13). Subsequently, a mixed solution containing EDTA (2 mM), Oxaloacetate (0.2 mM), Acetyl-CoA (0.14 mM), and DTNB (0.1 mM) in HEPES buffer was run through the membrane. The formation of 2-nitro-5-thiobenzoate (**TNB**, λ_max_ = 412 nm) was observed by the solution’s color change that occurred while passing the supramolecular membrane.

# Leaching of CS

Enzymes immobilized non-covalently (i.e. via adsorption or entrapment) on a porous support are prone to leach out of this support over time.[5] The amount of leaching enzymes and their effect on the biocatalytic reaction was assessed by the enzymatic activity of the filtrate fraction of neat buffer solution, which was run over immobilized enzyme on the **PP2b** supramolecular membrane (see section S12). For this, the reaction kinetics of undiluted buffer filtrate was measured as described in section S11, and compared to the enzymatic activity of unfiltered CS and unfiltered buffer solution (Figure S12). The activity of leaching enzymes is 0.23% with respect to the feed solution that was used for enzyme deposition (Table S4). The activity of leaching enzymes is small compared to the rapid color change that takes place during flow over immobilized enzymes in the supramolecular membrane (Figure S13).

**
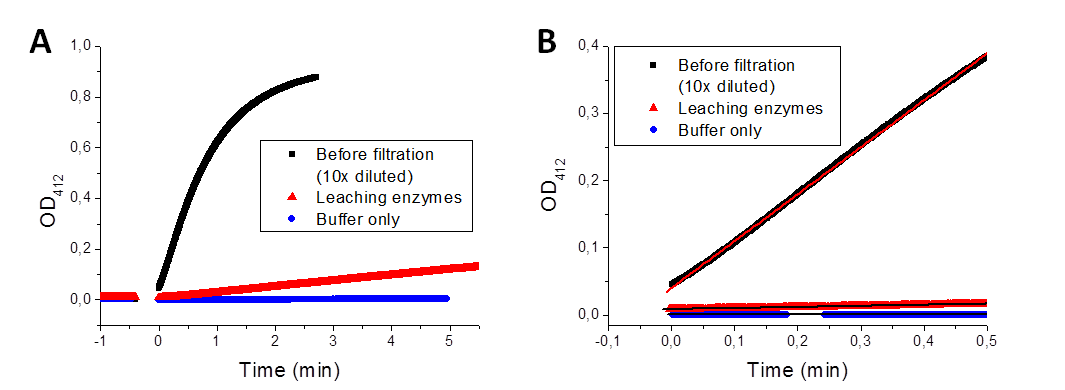
**

**Figure S12. Leaching of CS.** Kinetics of the activity of leaching CS, as compared to the activity of CS before filtration (10x diluted), and of neat buffer solution. (A) Full experiment. (B) Linear range of enzyme kinetics and regression lines.

**Table S4.** Slope of the linear fits (ΔOD_412_ / Δt) in the initial CS-catalyzed reaction kinetics, and relative enzyme activities.

|  | **ΔOD_412_ / Δt [min^-1^]** | **Relative activity [%]** |
| --- | --- | --- |
| **Before filtration** | - | 100 |
| **Before filtration (10x diluted)** | 0.6990 | 10 |
| **Leaching enzymes** | 0.0162 | 0.23 |
| **Buffer only** | 0.0004 | 0 |

**
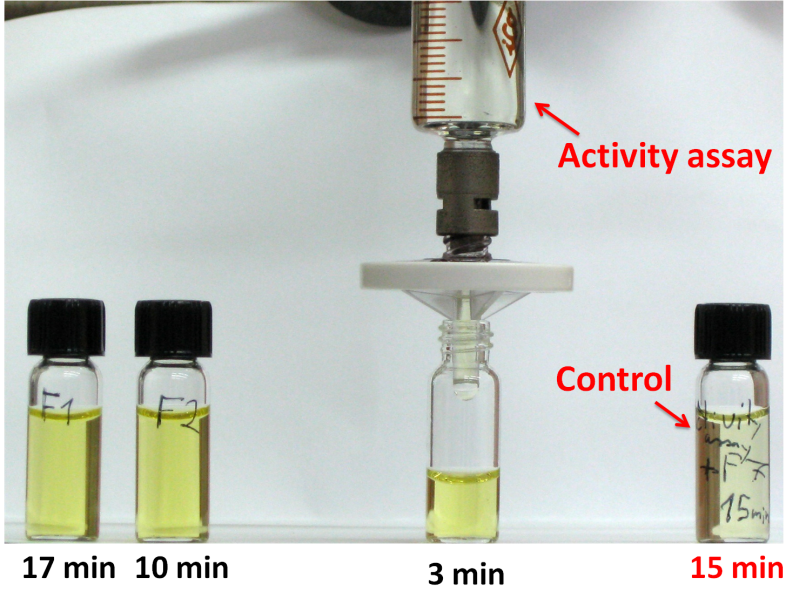
**

**Figure S13. Enzymatic activity of CS immobilized in the supramolecular membrane.** The photograph shows activity assay filtrate fractions F1, F2, and F3 (vials from left to right), which were collected successively. Elapsed times from the beginning of the collection of each of these fractions are specified. The collected fractions have identical color, showing that no further reaction takes place in solution. For comparison, a control sample of the filtrate of neat buffer solution is shown, to which the activity assay components were added only after filtration, and which was aged for 15 min (right vial). The control sample develops some yellow color after that time, indicating some activity of leaching enzymes. However, the conversion in solution is slow compared to the rapid color change, which is observed for the activity assay that was run through the membrane.

# β-Gal immobilization and biocatalysis

All experiments were conducted at a constant temperature of 20°C. For enzyme immobilization, a solution of β-Gal (1.5 ml, 0.2 mg/ml) in HEPES buffer was filtered over a freshly prepared supramolecular membrane. Subsequently, 7.5 ml neat buffer solution was filtered, in order to rinse out traces of penetrating enzymes. The filtrate of the buffer solution was collected, in order to assess the amount of leaching enzymes before the flow experiment (L_before_, see section S15). Subsequently, a solution of o‑nitrophenyl-β-D-galactoside (**ONPG**; 0.05 mg/ml) in Z-Buffer was filtered through the membrane. The biocatalysed hydrolysis of ONPG into Galactose and o-Nitrophenol (**ONP**, λ_max_ = 420 nm) was indicated by the yellow color of the filtrate.[1] The filter was connected to a UV/Vis flow cell in a spectrophotometer via PTFE tubing, and the absorbance at 420 nm (OD_420_) of the filtrate was recorded as a function of time, demonstrating stable conversion of ONPG into ONP during three hours of continuous operation (Figure S14). During that time approximately 29 ml of substrate solution had passed the membrane. Subsequently, addition of substrate solution was stopped, and neat buffer solution (12 ml) was filtered, in order to remove remaining substrate from the membrane and filter housing. The last fraction of this filtrate (1.5 ml) was collected separately, in order to evaluate enzyme leaching after the flow experiment (L_after_, see section S15). Altogether, there was a continuous flow of solvent over the membrane and stable performance for approximately 6 hours (including. membrane preparation, enzyme deposition, washing and heterogeneous biocatalysis).

**Figure S14. β-Gal biocatalysis.** Absorbance at 420 nm (OD_420_) as a function of time. Stable conversion of ONPG into ONP is observed during several hours of continuous flow of substrate. Instability of the absorbance at t ≈ 170 min was caused by an air bubble in the flow cell.

In order to determine the yield of the ONPG conversion, several samples of ONPG of different concentrations (0.005 ‑ 0.1 mg/ml) in Z-Buffer were quantitatively converted into ONP by addition of β-Gal (final concentration: 0.2 mg/ml) followed by aging for 40 min. The samples were injected successively into the UV/Vis flow cell, and OD_420_ was recorded. A calibration line was obtained by plotting OD_420_ vs. concentration (Figure S15). The plot shows excellent agreement with the Beer-Lambert law up to a concentration of 0.05 mg/ml. Based on the regression line slope (1.36 ml mg^-1^), the absorbance at 420 nm (OD_420, max_) of ONP produced quantitatively from 0.05 mg/ml ONPG is 0.068. With OD_420_ ≈ 0.061 measured during the continuous flow reaction (Figure S14), the average ONP yield (OD_420_ / OD_420, max_) was determined to be 90%.

**Figure S15. Calibration line for quantification of ONP.** OD_420_ vs. initial concentration of fully converted ONPG.

# Leaching of β-Gal

The amount of leaching β-Gal was assessed via the enzymatic activity of the filtrate fractions of neat buffer solution, that were collected immediately before (L_before_) and after (L_after_) the continuous flow experiment (section S14). For this, the OD_420_ of the test solution (1125 μl) was recorded for several minutes. Then a freshly prepared solution of ONPG (4 mg/ml) in Z-Buffer (375 μl) was added and the kinetic measurement was continued. The slope of OD_420_ (ΔOD_420_ / Δt) in the linear region of the kinetics plot (Figure S16) is proportional to the enzymatic activity under saturation condition. The relative activities are detailed in Table S5. Leaching enzyme activity was very low, ranging from 0.24% before the continuous flow experiment to 0.046% afterwards.

**Figure S16. Leaching of β-Gal.** Kinetics of the activity of leaching β-Gal (L_before_, L_after_), as compared to the activity of the β-Gal feed solution that was used for enzyme immobilization (10x diluted), and of neat buffer solution.

Table S5. Slope of the linear fits (ΔOD_420_ / Δt) in the initial β-Gal-catalyzed reaction kinetics, and relative enzyme activities.

|  | **ΔOD_420_ / Δt [min^-1^]** | **Relative activity [%]** |
| --- | --- | --- |
| **β-Gal feed solution** | **-** | 100 |
| **β-Gal feed solution (10x diluted)** | 0.3474 | 10 |
| **L_before_** | 0.0084 | 0.24 |
| **L_after_** | 0.0016 | 0.046 |
| **Background reaction** | ≤ 0.0001 | 0 |

# References

1. Miller JH (1972) Experiments in molecular genetics. New York: Cold Spring Harbor Laboratory.

2. Röthlisberger D, Khersonsky O, Wollacott AM, Jiang L, DeChancie J, et al. (2008) Kemp elimination catalysts by computational enzyme design. Nature 453: 190-195.

3. Bradford MM (1976) A rapid and sensitive method for the quantitation of microgram quantities of protein utilizing the principle of protein-dye binding. Anal Biochem 72: 248-254.

4. Srere PA, Brazil H, Gonen L (1963) The Citrate Condensing Enzyme of Pigeon Breast Muscle and Moth Flight Muscle. Acta Chem Scand 17: S129-S134.

5. Tran DN, Balkus KJ (2011) Perspective of Recent Progress in Immobilization of Enzymes. ACS Catal 1: 956-968.
